# Making clinician-scientists visible: methods for identifying clinician research participation and metrics at scale

**DOI:** 10.3389/frhs.2026.1791235

**Published:** 2026-04-30

**Authors:** Alicia Feldman, Henry Cook, Paula McDonald, Christina Malatzky, Peter Frederiksen

**Affiliations:** 1 Faculty of Business and Law, Queensland University of Technology, Brisbane, QLD, Australia; 2 Faculty of the Arts, Social Sciences and Humanities, University of Wollongong, Wollongong, NSW, Australia; 3 Faculty of Health, Queensland University of Technology, Brisbane, QLD, Australia

**Keywords:** clinician-scientists, data linkage, health services research, health workforce, research capacity, research workforce, workforce intelligence, bibliometrics

## Abstract

**Background:**

Clinician-scientists play a vital role in advancing health and medical research and health system innovation, yet their engagement in research, alongside their clinical role, is rarely measured in a systematic or comprehensive way. This longstanding gap in clinical workforce intelligence limits the ability of health systems to monitor research activity, design appropriate support for clinician-scientist career pathways, and allocate research resources effectively. This study aimed to develop and apply scalable methods to identify clinician-scientists and quantify their research contributions at a national level.

**Methods:**

This article presents novel and scalable data linkage methods for identifying clinician-scientists, and quantifying their scholarly contributions and research impact, by linking national medical registration data with data derived from Scopus Author Profiles. The approach identifies research-active clinicians (clinician-scientists) and generates verified research metrics for each individual. The methods were applied to medical practitioners in one national jurisdiction.

**Results:**

Applied to the full population of practising Australian medical practitioners (*n* = 134,701), the methods identified 15,933 (11.83%) medical practitioners with Scopus Author Profiles and yielded the first national dataset of verified research metrics for a clinician-scientist workforce. The method supports disaggregation by key demographic and professional characteristics, enabling detailed analysis across professional registration categories, research type and scale, individual variables, and institutional affiliations. The resulting dataset facilitates insights into the distribution, scale, and impact of clinician research, as well as the extent of research collaboration and equity.

**Conclusions:**

This study presents a transferable and scalable solution to a longstanding evidence gap in health workforce research. Systematically identifying clinician-scientists and quantifying their research activity can enhance the visibility of this workforce, support data-driven investment, and inform policies designed to strengthen research capability and capacity within health systems. The methods are adaptable to other clinical workforces, jurisdictions, and data sources, with clear implications for institutional reform and international collaboration. Improving the visibility of clinician-scientists offers opportunities to strengthen the clinician-scientist career pipeline, inform inclusive, data-driven health research policy, and support strategic investment in developing the research capability of the health workforce.

## 1 Introduction

Research undertaken by clinicians is a critical but under-monitored aspect of many health systems (1–5). Unlike health and medical research (HMR) generated in higher education, which is systematically tracked through institutional repositories and global bibliometric platforms, the research contributions of health practitioners (practising clinicians) are largely obscured and can go unnoticed (2, 5). This disconnect persists despite a growing body of evidence suggesting that new scientific knowledge generated by clinicians in health service contexts contributes to improved patient outcomes, improved organisational performance, and health systems innovation (2, 6–8). Often referred to as clinician-scientists (also clinician-researchers or clinician-investigators), these professionals bridge clinical practice and research, enabling the integration of evidence into care and the identification of relevant areas of inquiry. Although clinician-scientists play a key role in translating research into practice and shaping health system priorities (1, 9), their contributions are rarely captured or measured systematically (10, 11).

This article addresses this critical evidence gap by presenting a novel and replicable set of methods for identifying clinician-scientists and quantifying their research metrics at institutional, regional, national or multinational levels. In order to demonstrate the validity of these methods, we apply them to a complete national sample of Australian medical practitioners to identify physician-scientists (a label often used specifically for medically qualified clinician-scientists) and their research metrics. We link medical practitioner data from Australia's national registration agency and data derived from Elsevier's research repository, Scopus. The methods, and our resulting dataset, enable detailed analysis of the characteristics of practitioners who have Author Profiles indexed in Scopus, as well as the scope, scale and key metrics of the research they generate. The methods also allow for disaggregation by demographic variables including medical specialty, sex and geographic region. While the approach described in this article focuses on medically-qualified clinician-scientists (i.e., physician-scientists) in Australia, it could also be applied to other registered health practitioners such as nurses or psychologists, and to other health systems, including at institutional, regional, or international levels. To the best of our knowledge, this is the first study to apply a systematic set of methods that can identify which clinicians are (and are not) engaged in traditional research (indexed in Scopus), the types of research in which they participate, and the extent and impact of their contributions.

The article firstly synthesises the literature addressing the dual challenges of integrating research into clinical health settings in order to improve health outcomes, and measuring the extent and impact of research undertaken by clinician-scientists. The methods are then described, including the key variables constituting the two primary data sets, and the procedures used to link these data sources. Following the presentation of results, the article concludes with a discussion of the utility, limitations and broader potential applications of this new and innovative approach.

### 1.1 Facilitating and measuring research in clinical settings

Although health professionals are encouraged to engage with research evidence generation, health systems often lack mechanisms to make this activity visible at workforce scale (10). Systematically identifying clinician-scientists is complicated by the fragmented and decentralised nature of health workforce and research information systems—often governed by separate institutions with differing mandates and priorities. Clinicians often publish under varying institutional affiliations, name formats, or practice locations, and traditional research tracking systems are designed around university-employed academics rather than health service-based practitioners. As a result, an unknown but potentially large proportion of practising clinicians who generate peer-reviewed research remain invisible in health workforce intelligence systems. Such data gaps not only obstruct the identification, support, and evaluation of clinician-scientists but also impede efforts to ensure equity in research participation and institutional representation (4, 5, 12, 13). Considering that only 60% of healthcare is aligned with current evidence (14), and that clinical research activity correlates with lowered mortality and improved care quality (6, 15), addressing this data deficiency is essential to strengthening health systems and closing the translational gap (16, 17).

The lack of reliable, large-scale research data on the clinician-scientist workforce is particularly troubling considering that although there are growing calls for research to be embedded with healthcare to drive quality and innovation (2, 18, 19), there are growing concerns that the clinician-scientist workforce is in decline globally (5, 20–23). Growing pressures within health systems to become more efficient and sustainable (including to better manage costs and demand for services) have pushed research to the periphery of clinical practice, making it increasingly difficult for clinicians to sustain dual roles (2, 5, 16, 19, 22, 24). Sustainable models of integrating research into frontline care depend on the ability to identify, support, and evaluate the research undertaken by clinician-scientists and to quantify the safety, quality and efficiency value of this research activity to the service. Nevertheless, many face structural barriers to participating in and advancing their research, including limited protected time, inadequate funding, and unclear career pathways (16, 23). The situation is compounded by the fact that the clinician workforce is ageing and, in some professions and specialties and at leadership levels, under-representative of women and other groups who experience marginalisation in contemporary society (25, 26). This is of key concern, given that quality health outcomes depend on a health and social care workforce that reflects the diversity of the community it serves (13).

A further concern relates to clinicians’ access to, and participation in, formal research training. A recent U.S. report found that only 9.2% of physician-scientists hold a PhD (5), indicating that a large and often invisible cohort is conducting research without having progressed through established research training pathways. Although this survey was valuable in estimating a national prevalence of physician-scientists, its broad inclusion criteria and reliance on self-reported engagement limit the assessment of research activity and outputs. At a national level, the extent, nature, and distribution of clinicians’ research contributions are therefore difficult to evaluate through survey methods alone.

In Australia, data on research participation and research metrics of health practitioners remain incomplete. The Australian Academy of Health and Medical Sciences noted in their 2022 report *Research and Innovation as Core Functions in Transforming the Health System* (2) that clinician-scientists are:

*central to a workforce that can embed research in the health system. At present, we do not know how many clinician researchers there are in Australia, and they have no clear training pathway and face many barriers in pursuing this career path.*

The agency from which we acquired medical practitioner information—the Australian Health Practitioner Registration Agency (AHPRA)—distinguishes between roles that are clinical-dominant and research-dominant. However, it does not routinely collect or report information about research involvement for clinicians who identify as being primarily engaged in clinical work. Further, although the *Medicine in Australia: Balancing Employment and Life* (MABEL) survey (27) provided valuable longitudinal data on Australian doctors’ career trajectories, it did not track research participation or outputs. This lack of integrated data undermines efforts to build research capacity in the medical workforce, evaluate the investments of major national funding initiatives such as the Medical Research Future Fund (MRFF) and National Health and Medical Research Council (NHMRC), and the level of equity in research opportunities that exists across regions, specialties, and demographic groups. Without such data, national health systems cannot effectively assess progress toward embedding research within clinical care, or address the systemic barriers that discourage clinicians from pursuing or continuing research careers (22).

While there is no accepted set of activities that define clinician research participation and performance, clinician engagement in research often encompasses a range of intellectual and practical activities such as research design, undertaking clinical trials, data analysis, population/cohort health studies, quality assurance and audits, literature reviews to inform policy, and quality improvement and evidence translation activities (4, 15, 28). The present study operationalises research participation and performance at the output-generating end of this spectrum. Specifically, it identifies clinician-scientists as those with Scopus-indexed outputs. While this approach differs from studies that rely on self-report survey designs, our narrower scope does exclude clinicians engaged in other important research activity such as quality improvement, guideline development, supervision, or locally disseminated research. Our intent is to not suggest one or the other is more critical but instead highlight the affordances and method to surfacing a currently unrecorded expertise of clinician-scientists.

The methods presented in this article respond directly to these long-standing challenges in identifying and supporting clinician-scientists, providing a systematic, scalable, and replicable solution to critical data gaps using existing data sources. Our approach has the ability to enhance the visibility of clinician-scientists by quantifying and benchmarking research activity amongst clinicians across different health settings and monitoring which sub-groups are participating in and producing research. Equipped with such information, health leaders and policymakers can make more informed decisions about investments in health research and advance efforts to strengthen research capacity and more fully integrate research into clinical practice.

## 2 Data sources

Two primary datasets were utilised for the study; the registration data of medical practitioners and Scopus Author Profile records. Further details are provided below:

1. Medical Registration Data: The Australian Health Practitioner Regulation Agency (AHPRA) maintains a register of all practising health practitioners in Australia. AHPRA's role includes ensuring that registered practitioners are appropriately trained, qualified, and safe to practise. Only clinicians with an active AHPRA registration can practice in Australia. Data recorded by AHPRA for each registered individual comprises items such as name, health qualification, registration status, practice location, and specialist designation (see Online Supplementary 3 for an example of commonly collected fields). A single data file containing this information for each medical practitioner registered in Australia was purchased from AHPRA. The data was cleaned and transformed for our purpose, ensuring all registered individuals were represented across a single row, and multi-item fields (e.g., individuals with more than one qualification or specialty) were separated.
2. Scopus Author Profile records: Bibliometric data were obtained from Elsevier's Scopus database. Scopus is a comprehensive, international bibliographic data source, indexing over 70 million publication records (29). Elsevier provides various Application Programming Interfaces (APIs) to request bibliometric and non-traditional metrics data at the author, publication, institution, national, and global levels (30).

## 3 Methods

The following sections outline the procedural steps involved in linking the data sources. We also consider how the methods may be replicated in other health professions and contexts.

**Figure 1:**
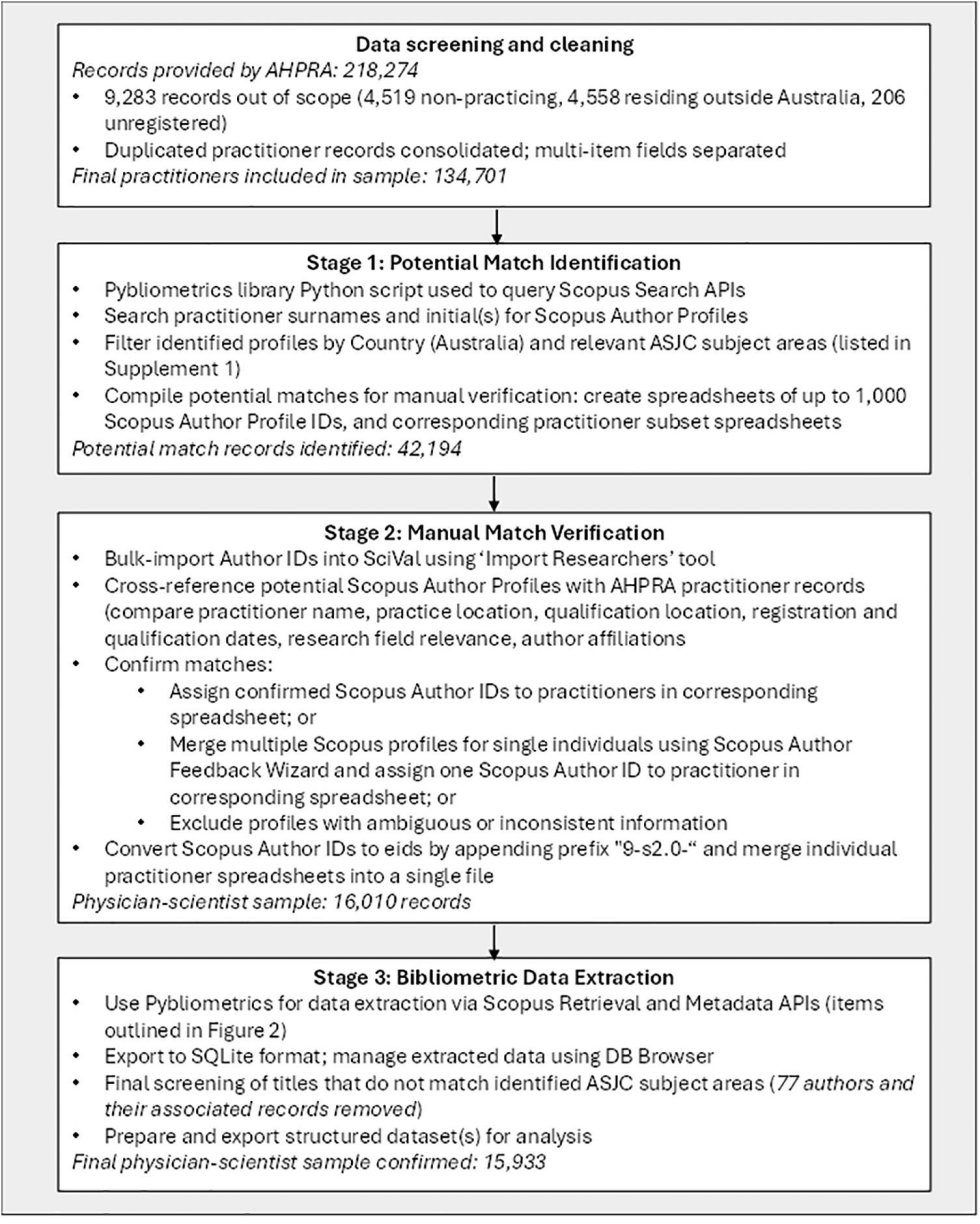
Methods for identifying clinician-scientists; Australian physician-scientist example.

A three-stage process was developed to match medical practitioners to corresponding Author Profiles in Scopus: potential match identification, manual match verification, and bibliometric data extraction. These steps are summarised in Figure 1 and are described in detail below.

Candidate authors were first identified using Python, a programming language suitable for handling data-orientated tasks. A dedicated script was developed that leveraged the Python library Pybliometrics (31) to query Scopus Search APIs (30). An API allows software to request specific data from an online service. In this case, the Pybliometrics library acted as an intermediary, sending structured queries to Scopus and retrieving matching author records in a format that could be processed automatically. This query determined whether a practitioner's name existed within the Scopus Author Profiles database. These identified Author Profiles were then filtered/narrowed based on country (Australia) and relevant All Science Journal Classification (ASJC) subject areas (as outlined in Online Supplementary 1). ASJC subject areas are standardised research categories that classify journals and their articles by discipline, enabling consistent grouping of research outputs. These refined results were considered potential matches with AHPRA-registered practitioners.

The returned potential matches were compiled for manual screening, which involved comparing each identified Scopus Author Profile against AHPRA records. Multiple relevant fields were cross-referenced, including names, practice location, qualification locations, registration and qualification dates, research field relevance, and author affiliations. To streamline the verification process, spreadsheets of Author IDs were uploaded via the SciVal ‘Import Researchers’ function, which enables the bulk import of up to 1,000 Scopus Author IDs at a time. SciVal is a web-based analytics platform developed by Elsevier that allows users to assess and visualise research performance using data from Scopus. In this context, the import tool was used to rapidly view author profiles at scale. For each imported set of Scopus Author IDs, a corresponding spreadsheet of AHPRA-registered practitioners with matching surnames was created to manage the manual comparison process more effectively.

Manual verification was conducted by five researchers. Prior to commencing independent matching, all team members completed a structured training exercise using a shared test set of 227 practitioners and their identified potential Author profiles. This was supported by a training manual constructed by the lead author detailing a range of matching scenarios and decision rules. Initial agreement on the training set was 98.2%, with discrepancies resolved through discussion to establish a common matching standard before independent work began. Consistency was further monitored through periodic spot checks, with 5% of potential matches independently reviewed by a second team member. No systematic discrepancies were identified during spot checking, confirming that the shared training protocol produced consistent application of the matching criteria. Author Profiles with inconsistent or ambiguous information were excluded rather than resolved by majority decision, ensuring that precision was prioritised. Where a Scopus Author Profile was deemed to match a practitioner record, the practitioner was assigned the corresponding Scopus Author ID. In some cases, multiple Scopus Author Profiles were identified as belonging to a single individual. This most commonly occurred where an individual published under multiple institutional affiliations and separate Author Profiles had not been automatically merged by the Scopus algorithm (29). These profiles (and therefore associated bibliometric and other data) were merged as part of the manual screening process, using the Scopus Author Feedback Wizard.

The final list of matched authors was used to populate a new Author eid column in the AHPRA dataset by appending the required prefix “9-s2.0-” to each verified Scopus Author ID. The eid (Electronic Identifier) is a unique identifier format used by Scopus to identify specific entities, distinguishing between entity types (e.g., prefix 9-s2.0- for authors, 2-s2.0- for publications). Using the correct eid prefix ensures compatibility with programmatic queries and functions via API and helps prevent entity misclassification during automated data retrieval.

**Figure 2:**
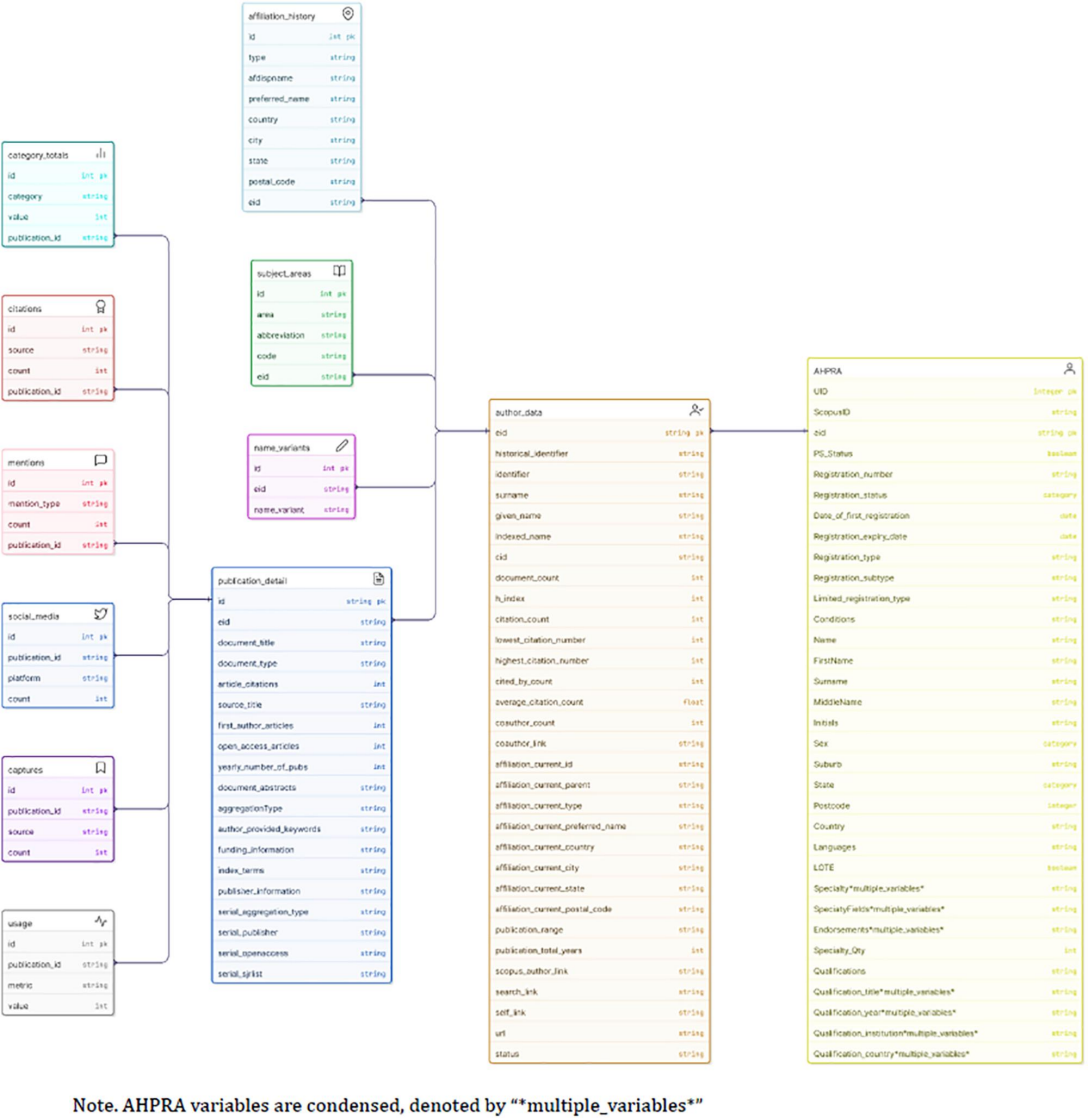
Entity relationship diagram.

Finally, the individual practitioner spreadsheets were merged, and relevant data points from Scopus Retrieval and Metadata APIs were extracted/collated via Pybliometrics for each verified Scopus Author ID. This data included detailed author metrics and publication metrics (see Figure 2 for a complete list). Extraction was managed using a relational database. Relational databases organise data into tables that can be connected (or “related”) through shared keys. This structure allows for flexible and reliable joining of information across multiple tables, enabling selected variables from the extracted bibliometric data to be efficiently linked with relevant fields from the original practitioner dataset at the time of analysis, while maintaining integrity of large complex datasets. This design supports reproducibility, scalability, and tailored linkage strategies to suit specific research questions, analytical needs and data visualisation. As a final accuracy check, article titles for all ASJC subject areas that were not identified in Online Supplementary 1 were reviewed to confirm inclusion in the dataset.

### 3.1 Replicability and adaptation

The methods are designed to be replicable across registries of licensed practitioners, are transferable to other health professions, and can be adapted to smaller-scale (e.g., health institution level) or larger-scale (e.g., multinational, multiple occupations) analyses. Minor adaptations may be required to accommodate local data structures, naming conventions, or registration systems.

Replication requires, at minimum, a practitioner registry containing full legal names, practitioner types, qualifications, registration dates, and practice location, alongside a bibliometric database with author-level identifiers and API access. Where practitioner names are common or registry data is limited, additional identifying fields such as institutional affiliation history, graduation year, or specialty improve matching precision and reduce manual verification time. Governance conditions will also shape feasibility. Formal data access agreements with the relevant registration authority and ethical approval should be anticipated.

We applied the methods to the entire population of Australian medical practitioners, which involved substantial resources. These resources included significant researcher time, specialised coding and data analysis skillsets, and substantial non-human resources. Since our application has provided proof-of-concept, future applications might consider using a stratified random sample from practitioner databases to improve feasibility while maintaining representativeness.

The method is most readily transferable to professions with centralised national registration systems where equivalent registration data exists. Institutional-level datasets such as employment records offer a viable alternative to national registry data and carry the added advantage of containing richer information about clinical position and activity, allocated research workload, remuneration/compensation, and all aspects of employee journey.

## 4 Results

Application of the methods yielded a national dataset of research-engaged medical practitioners in Australia. The initial AHPRA dataset included 218,274 records as of April 3rd, 2024. Of the 218,274 original records, 9,283 (4.25%) were removed as they did not fit the scope for the intended population (see Figure 1). A large number of duplicate entries, primarily due to separate general and specialist registration records, were subsequently combined, resulting in a sample of 134,701 registered Australian medical practitioners. Using the three-stage matching protocol described above, 15,933 medical practitioners were successfully linked to a Scopus Author Profile, representing 11.83% of the practising national medical workforce. These individuals had at least one indexed item in Scopus between 1996 and the application period (stages 1–3 were completed between June 2024 and August 2025). Given the manual verification processes and the use of a full population sample, this proportion is likely to be highly accurate. However, because a small number of practitioners were excluded due to inconsistent or ambiguous identity markers that could not be confidently verified, and taking into account Scopus coverage limitations, the estimate of physician-scientists as a proportion of the medical workforce is likely to be conservative rather than overstated.

The full dataset included 185 variables from which additional measures can also be constructed. Data was stored across 12 data tables (Figure 2). Due to the manual insertion of Scopus Author IDs and eids into the practitioner data table, all tables (and contained variables) can be linked via shared keys to support tailored analyses. Author eids connect author-related tables and publication eids connect publication-related tables. The comprehensive dataset lends itself to multiple areas of investigation across several themes including: clinician research participation and metrics; institutional and geographic trends; bibliometric productivity and impact; knowledge translation and non-traditional impact; equity and visibility gaps; and collaboration and career trajectory trends. Online Supplementary 2 outlines example research questions under each of these areas of investigation.

## 5 Discussion

The application of these methods to the full population of practising Australian medical practitioners provides the first empirically derived national estimate of clinician research participation based not on self-reported information but observed outputs. Our approach directly addresses longstanding evidence gaps highlighted by previous work, notably Willcox's (24) summary of previous attempts to estimate the size of clinician-scientist workforces, and Browne's (5) estimate of physician-scientist prevalence in the U.S. using self-report survey methods and broad inclusion criteria. Although the methodologies of our study and Browne's differ, the relatively similar prevalence figures of 14% in the U.S. and 11.83% in Australia are notable given the differences in national research training pathways and comparatively limited structural support for clinician-scientists in Australia (16, 32).

From a health workforce perspective, the development of these methods has several important implications. First, they demonstrate the feasibility of integrating large and separately governed data systems to construct research intelligence at the clinician level—a capacity that is currently absent from most national HMR infrastructures. Second, the ability to disaggregate data by specialty, sex, location, and other characteristics creates new opportunities for targeted workforce support and capacity building, and the ability to assess and respond to inequalities.

There is also relevance from an education and research training perspective. The ability to identify which clinicians produce Scopus-indexed outputs, and to link this to registration data including qualification type, graduation year, and specialty, creates new opportunities to examine how training pathways relate to sustained research activity. For example, the dataset could be used to assess whether completion of a research higher degree is associated with greater or more sustained research output, or to identify the career stages when clinicians are most likely to enter or exit research activity. This has practical implications for the design of education and training programs, enabling evaluation of whether these investments produce sustained clinician research careers or concentrated output that diminishes over time. The dataset also provides a basis for examining supervision capacity across the workforce, identifying practitioners with the publication records and career trajectories that would support supervision and mentoring of aspiring clinician-scientists. At a system level, these insights can inform decisions about where to direct research training resources and how to better support the integration of research into clinical practice.

The findings also highlight the structural invisibility of many research-active clinicians within current health workforce intelligence systems. During the manual screening stage, we noted that many of the researchers that required multiple profile linking held dual roles in clinical and academic settings, while others appeared to produce research outputs entirely from within health service settings. This underscores the limitations of research monitoring systems that are oriented primarily around university employment and reinforces the need for whole of workforce approaches that recognise research as an activity performed across organisational and sector boundaries. By identifying research-active clinicians, health systems and funders can design more inclusive support schemes, track progress in broadening participation, benchmark institutional, regional or local research contributions and ultimately better measure the contribution of research to safety, quality and efficiency of healthcare at the system level.

While the methods proved viable, several limitations warrant discussion. The accuracy of the linkage process depends substantially on the completeness and consistency of both registration and bibliometric records. Disambiguating common names was a significant challenge, even with manual verification. The fragmentation of Author Profiles within Scopus also introduces complexity, particularly for practitioners with research outputs across numerous profiles that result from name variations and/or different institutional affiliations. This is more challenging in cases where multiple practitioners share the same first and last name. The study excluded a limited number of potential authors with inconsistent or ambiguous identity markers to avoid false positives. This conservative approach ensured high confidence in the matched sample but likely resulted in undercounting. Hence, the final dataset should be interpreted as a lower-bound estimate. Mitigating this limitation however, we classified all practitioners as clinician-scientists if they had a verified Scopus Author Profile. This meant that those who had engaged in research in the past, but were no longer engaged, were included in the initial prevalence figure. Indeed, evidence suggests that many clinician-scientists are stepping back from dual roles (9, 33, 34). The definition of clinician-scientist used for the study was also constrained by the approach and available data. The automated filtering of potential matches by country and ASJC subject area was necessary for study feasibility but may have excluded a small number of individuals from the population of interest. While these filters were applied in the screening stage, extraction was not constrained by these filters. Also, author metrics collected are limited only to items indexed by Scopus and although extensive, Scopus may also underrepresent research outputs in specific fields or specialties, particular forms of scholarship, or outputs published in regional or non-English journals (35). As such, this method may underestimate research engagement in certain areas. Taking these caveats together, our prevalence figure likely reflects a conservative estimate of both current and past Australian physician-scientists.

Formal research outputs indexed in Scopus and captured in this study do not represent the full range of research activity, engagement and contributions. Many clinicians contribute to research through, for example, quality improvement activities, supervision, development of clinical guidelines, conference presentations, and grant schemes not indexed by Scopus. Additionally, structural biases in academic publishing, including a well-documented tendency to favour statistically significant or novel results, mean that valuable contributions to research may go unpublished or unrecognised in formal citation databases. Consequently, our method identifies only a subset of the broader research-engaged workforce. Collecting and incorporating a broader scope of research engagement activities would strengthen understandings of practitioner research participation and metrics.

In its current form, this method is a proof-of-concept. The reliance on a full population sample and resource-intensive manual verification may limit routine implementation. Without adjusting the sampling approach, this limitation could most effectively be addressed by improving the data infrastructure that supports clinician research visibility, and via the collection of unique identifiers by registration bodies such as AHPRA. Persistent unique identifiers already embedded in research information systems, such as ORCIDs, Scopus Author IDs, or PubMed Author IDs, at the point of practitioner registration and renewal, would allow for more automated and scalable tracking of clinician research engagement over time. This relatively simple requirement could be further strengthened by the establishment of a national coordinating body with responsibility for managing clinician-scientist data, ideally supported through collaboration between health and higher education sectors. Short of this, targeted awareness campaigns to promote the uptake and maintenance of Open Researcher and Contributor ID (ORCID) profiles by clinicians could offer a simple, cost-effective, high-impact alternative. ORCID IDs are free, globally recognised researcher identifiers that can be linked to multiple bibliometric platforms, ensuring continuity and discoverability of individual research contributions over time (36). Beyond ensuring precision in the current study, the manual verification process has generated a validated dataset that could be used to train and benchmark AI-assisted classification models (using supervised machine learning approaches), enabling future iterations to operate at scale with reduced human oversight.

The dataset linking process used in this study opens multiple avenues for further research. The approach could be used to track longitudinal patterns in clinician-scientist engagement, analyse productivity by demographic and professional characteristics, examine the relationship between research involvement and health system outcomes, and evaluate institutional or policy interventions or investments aimed at supporting clinical research careers. The methods could also be adapted to other registered health professions to provide insights into interdisciplinary research engagement. Browne's (5) finding that only a minority of physician-scientists in the U.S. hold a PhD further underscores the need to better understand the characteristics and trajectories of clinician-scientists outside traditional academic training pathways. Applying and extending these methods to other jurisdictions would also enable more rigorous cross-country comparisons and provide a foundation for more effectively evaluating the impact of specific interventions such as MD-PhD programs, targeted fellowships, and research capacity-building initiatives such as mentoring programs (37). Finally, technical enhancements such as the integration of machine learning tools for author disambiguation and the use of additional research and workforce databases—ideally coupled with institutional reform—may improve completeness and simplify replication.

This study addressed a critical gap in current understanding of the clinician research workforce by developing and testing novel and scalable methods for systematic identification of clinician-scientists and quantification of their research metrics. By matching national physician registration records with Scopus Author Profiles, we generated a single dataset that identifies, with a very high level of precision, the prevalence of physician-scientists within the medical workforce and the scale and distribution of their research metrics. The methods are adaptable to other health professions, jurisdictions, and data sources, and offer potential for international comparisons and benchmarking of clinician research capacity. Future implementation would be strengthened through data infrastructure reforms, particularly the routine collection of persistent researcher identifiers within practitioner registration systems. Such developments would support more efficient monitoring, reduce reliance on manual verification, and enable longitudinal tracking of clinician research engagement. Our approach, while imperfect, makes clinician-scientists visible at scale. It positions research as a core component of the health workforce and of healthcare activity, offering new and significant opportunities for data-driven policy, support, investment and decision-making in health systems globally.

## Data Availability

The datasets presented in this article are not readily available because the data from this study cannot be made publicly available. Requests to access the datasets should be directed to Alicia Feldman, a.feldman@qut.edu.au.

## References

[1] Ponka D Coffman M Fraser-Barclay KE Fortier RDW Howe A Kidd M Fostering global primary care research: a capacity-building approach. BMJ Glob Health. (2020) 5:e002470. 10.1136/bmjgh-2020-002470 32624501 PMC7337619

[2] Australian Academy of Health and Medical Sciences. Research and Innovation as Core Functions in Transforming the Health System: A Vision for the Future of Health in Australia. Canberra, ACT: Australian Academy of Health and Medical Sciences (2022). Available online at: https://aahms.org/wp-content/uploads/2022/10/AAHMS-Vision-Report.pdf (Accessed May 27, 2025).

[3] Kengia JT Kalolo A Barash D Chwa C Hayirli TC Kapologwe NA Research capacity, motivators and barriers to conducting research among healthcare providers in Tanzania’s public health system: a mixed methods study. Hum Resour Health. (2023) 21:73. 10.1186/s12960-023-00858-w 37670321 PMC10478476

[4] Yoong SL Bolsewicz K Reilly K Williams C Wolfenden L Grady A Describing the evidence-base for research engagement by health care providers and health care organisations: a scoping review. BMC Health Serv Res. (2023) 23:75. 10.1186/s12913-022-08887-2 36694193 PMC9872336

[5] Browne A. Prevalence and characteristics of physicians engaged in research in the US. JAMA Netw Open. (2024) 7:e2433140. 10.1001/jamanetworkopen.2024.33140 39316404 PMC11423164

[6] Ozdemir BA Karthikesalingam A Sinha S Poloniecki JD Hinchliffe RJ Thompson MM Research activity and the association with mortality. PLoS One. (2015) 10:e0118253. 10.1371/journal.pone.0118253 25719608 PMC4342017

[7] Greenhalgh T Raftery J Hanney S Glover M. Research impact: a narrative review. BMC Med. (2016) 14:78. 10.1186/s12916-016-0620-8 27211576 PMC4876557

[8] Vindrola-Padros C Pape T Utley M Fulop NJ. The role of embedded research in quality improvement: a narrative review. BMJ Qual Saf. (2017) 26:70. 10.1136/bmjqs-2015-004877 27129492 PMC5256405

[9] Goldstein AM Blair AB Keswani SG Gosain A Morowitz M Kuo JS A roadmap for aspiring surgeon-scientists in today’s healthcare environment. Ann Surg. (2019) 269:66–72. 10.1097/SLA.0000000000002840 29958227 PMC6298819

[10] Finn M Gilmore B Sheaf G Vallières F. What do we mean by individual capacity strengthening for primary health care in low- and middle-income countries? A systematic scoping review to improve conceptual clarity. Hum Resour Health. (2021) 19:5. 10.1186/s12960-020-00547-y 33407554 PMC7789571

[11] Hughes TL George M Shah R Dias BM Dohrn JE Bortoli Cassiani D Nursing engagement in research priorities focused on health systems and services in Latin America countries. Hum Resour Health. (2022) 20:45. 10.1186/s12960-022-00746-9 35606802 PMC9125952

[12] Traill CL Januszewski AS Larkins RG Keech AC Jenkins AJ. Time to research Australian female physician-researchers. Intern Med J. (2016) 46:412–9. 10.1111/imj.12986 26692102

[13] Kelly-Blake K Garrison NA Fletcher FE Ajegba B Smith N Brafford M Rationales for expanding minority physician representation in the workforce: a scoping review. Med Educ. (2018) 52:925–35. 10.1111/medu.13618 29932213

[14] Runciman WB Hunt TD Hannaford NA Hibbert PD Westbrook JI Coiera EW Caretrack: assessing the appropriateness of health care delivery in Australia. Med J Aust. (2012) 197:100–5. 10.5694/mja12.10510 22794056

[15] Boaz A Hanney S Jones T Soper B. Does the engagement of clinicians and organisations in research improve healthcare performance: a three-stage review. BMJ Open. (2015) 5:e009415. 10.1136/bmjopen-2015-009415 26656023 PMC4680006

[16] Mills JMZ Januszewski AS Robinson BG Traill CL Jenkins AJ Keech AC. Attractions and barriers to Australian physician-researcher careers. Intern Med J. (2019) 49:171–81. 10.1111/imj.14086 30152020

[17] Group of 8 Australia (2020). Strengthening Australian Clinical Research – Group of Eight Submission to the Medical Workforce Reform Advisory Committee. Available online at: https://go8.edu.au/wp-content/uploads/2020/12/Go8-AICRTP-FINAL.pdf (Accessed March 13, 2025).

[18] Braithwaite J Mannion R Matsuyama Y Shekelle PG Whittaker S Al-Adawi S The future of health systems to 2030: a roadmap for global progress and sustainability. Int J Qual Health Care. (2018) 30:823–31. 10.1093/intqhc/mzy242 30576556

[19] Academy of Medical Sciences. Future-proofing UK Health Research: A People-centred, Coordinated Approach. London: Academy of Medical Sciences (2023). Available online at: https://acmedsci.ac.uk/file-download/23932583 (Accessed June 7, 2025).

[20] Wyngaarden J. The clinical investigator as an endangered species. N Engl J Med. (1979) 301(23):1254–9. 10.1056/NEJM19791206301230 503128

[21] Culliton BJ. Clinical investigation: an endangered science. Nat Med. (1995) 1:281. 10.1038/nm0495-281 7585048

[22] Primack BA Dilmore TC Switzer GE Bryce CL Seltzer DL Li J Brief report: burnout among early career clinical investigators. Clin Transl Sci. (2010) 3:186–8. 10.1111/j.1752-8062.2010.00202.x 20718821 PMC2925292

[23] Bechtold JE Williams BR Weinstein SL Polly DW Pugely AJ Buckwalter JA The pursuit of scholarship: why we should care about resident research. J Bone Joint Surg Am. (2017) 99:e119. 10.2106/JBJS.16.01502 29135674

[24] Willcox S. Creating and Sustaining the Next Generation of the Clinical Academic Workforce: Issues and Strategies for Australia and New Zealand. Sydney, NSW: Medical Deans Australia and New Zealand (2011). Available online at: https://medicaldeans.org.au/md/2018/07/201108-Clinical-Academic-Workforce-DP-19-Aug-2011.pdf (Accessed March 15, 2025).

[25] Australian Medical Association. Diversity & Inclusion Plan 2020-2022. Barton, ACT, Australia: Australian Medical Association (2020). Available at: Available online at: https://www.ama.com.au/sites/default/files/documents/AMA_Diversity_%2526_Inclusion_Plan_V4_%25281%2529.pdf (Accessed March 24, 2023).

[26] Carethers JM. Toward realizing diversity in academic medicine. J Clin Invest. (2020) 130:5626–8. 10.1172/JCI144527 32990684 PMC7598036

[27] Joyce CM Scott A Jeon S-H Humphreys J Kalb G Witt J The “medicine in Australia: balancing employment and life (MABEL)” longitudinal survey - protocol and baseline data for a prospective cohort study of Australian doctors’ workforce participation. BMC Health Serv Res. (2010) 10:50. 10.1186/1472-6963-10-50 20181288 PMC2837653

[28] Hanney S Boaz A Jones T Soper B. Engagement in research: an innovative three-stage review of the benefits for health-care performance. Health Serv Deliver Res. (2013) 1:1–152. 10.3310/hsdr01080 25642550

[29] Elsevier Research Intelligence (2019). Research Metrics Guidebook. Available online at: https://supportcontent.elsevier.com/RightNow%20Next%20Gen/SciVal/ACAD_RL_ElsevierResearchMetricsBook_WEB.pdf (Accessed June 14, 2025).

[30] Rose ME Kitchin J. Pybliometrics API Reference. (2024). Available online at: https://pybliometrics.readthedocs.io/en/stable/reference.html (Accessed May 6, 2024).

[31] Rose ME Kitchin JR. Pybliometrics: scriptable bibliometrics using a python interface to scopus. SoftwareX. (2019) 10:100263. 10.1016/j.softx.2019.100263

[32] Eley DS. The clinician-scientist track: an approach addressing Australia’s need for a pathway to train its future clinical academic workforce. BMC Med Educ. (2018) 18(1):227. 10.1186/s12909-018-1337-5 30285826 PMC6171239

[33] Baumal R Benbassat J Van JAD. Reflections on the current and future roles of clinician-scientists. Israel Medl Assoc J. (2014) 16:475–8. PMID: 25269336

[34] Jain MK Cheung VG Utz PJ Kobilka BK Yamada T Lefkowitz R. Saving the endangered physician-scientist — a plan for accelerating medical breakthroughs. N Engl J Med. (2019) 381:399–402. 10.1056/NEJMp1904482 31365796

[35] Dresbeck R. Scival. J Med Libr Assoc. (2015) 103:164–6. 10.3163/1536-5050.103.3.018

[36] ORCID Support (2019). What is my ORCID iD and how should I use it? Available online at: https://support.orcid.org/hc/en-us/articles/360006897334-What-is-an-ORCID-iD-and-how-do-I-use-it (Accessed June 06 2025).

[37] Toyin-Thomas P Wariri O Ikhurionan P. Harnessing peer mentorship as a tool to turn human resource for health brain drain into brain gain: a case study of a Nigerian peer-mentored research group. Hum Resour Health. (2024) 22:46. 10.1186/s12960-024-00932-x 38937753 PMC11212419

