# Supplementary material for: Making clinician-scientists visible: methods for identifying clinician research participation and metrics at scale
Source: Front Health Serv. 2026 Apr 30;6:1791235. doi: 10.3389/frhs.2026.1791235 (PMC13171505; doi:10.3389/frhs.2026.1791235)
Supplement: Supplementary file 1 [file Supplementaryfile1.docx]

## Supplementary One: ASJC inclusion items

| **Code** | **Description** |
| --- | --- |
| Multidisciplinary | |
| 1000 | Multidisciplinary |
| Biochemistry, Genetics and Molecular Biology | |
| 1300 | General Biochemistry, Genetics and Molecular Biology |
| 1301 | Biochemistry, Genetics and Molecular Biology (miscellaneous) |
| 1302 | Aging |
| 1303 | Biochemistry |
| 1304 | Biophysics |
| 1305 | Biotechnology |
| 1306 | Cancer Research |
| 1307 | Cell Biology |
| 1308 | Clinical Biochemistry |
| 1309 | Developmental Biology |
| 1310 | Endocrinology |
| 1311 | Genetics |
| 1312 | Molecular Biology |
| 1313 | Molecular Medicine |
| 1314 | Physiology |
| 1315 | Structural Biology |
| Chemical Engineering | |
| 1502 | Bioengineering |
| Engineering | |
| 2204 | Biomedical Engineering |
| Environmental Science | |
| 2307 | Health, Toxicology and Mutagenesis |
| Immunology and Microbiology | |
| 2400 | General Immunology and Microbiology |
| 2401 | Immunology and Microbiology (miscellaneous) |
| 2402 | Applied Microbiology and Biotechnology |
| 2403 | Immunology |
| 2404 | Microbiology |
| 2405 | Parasitology |
| 2406 | Virology |
| Materials Science | |
| 2502 | Biomaterials |
| Medicine | |
| 2700 | General Medicine |
| 2701 | Medicine (miscellaneous) |
| 2702 | Anatomy |
| 2703 | Anesthesiology and Pain Medicine |
| 2704 | Biochemistry (medical) |
| 2705 | Cardiology and Cardiovascular Medicine |
| 2706 | Critical Care and Intensive Care Medicine |
| 2707 | Complementary and alternative medicine |
| 2708 | Dermatology |
| 2709 | Drug Guides |
| 2710 | Embryology |
| 2711 | Emergency Medicine |
| 2712 | Endocrinology, Diabetes and Metabolism |
| 2713 | Epidemiology |
| 2714 | Family Practice |
| 2715 | Gastroenterology |
| 2716 | Genetics (clinical) |
| 2717 | Geriatrics and Gerontology |
| 2718 | Health Informatics |
| 2719 | Health Policy |
| 2720 | Hematology |
| 2721 | Hepatology |
| 2722 | Histology |
| 2723 | Immunology and Allergy |
| 2724 | Internal Medicine |
| 2725 | Infectious Diseases |
| 2726 | Microbiology (medical) |
| 2727 | Nephrology |
| 2728 | Neurology (clinical) |
| 2729 | Obstetrics and Gynecology |
| 2730 | Oncology |
| 2731 | Ophthalmology |
| 2732 | Orthopedics and Sports Medicine |
| 2733 | Otorhinolaryngology |
| 2734 | Pathology and Forensic Medicine |
| 2735 | Pediatrics, Perinatology and Child Health |
| 2736 | Pharmacology (medical) |
| 2737 | Physiology (medical) |
| 2738 | Psychiatry and Mental health |
| 2739 | Public Health, Environmental and Occupational Health |
| 2740 | Pulmonary and Respiratory Medicine |
| 2741 | Radiology, Nuclear Medicine and imaging |
| 2742 | Rehabilitation |
| 2743 | Reproductive Medicine |
| 2744 | Reviews and References (medical) |
| 2745 | Rheumatology |
| 2746 | Surgery |
| 2747 | Transplantation |
| 2748 | Urology |
| Neuroscience | |
| 2800 | General Neuroscience |
| 2801 | Neuroscience (miscellaneous) |
| 2802 | Behavioral Neuroscience |
| 2803 | Biological Psychiatry |
| 2804 | Cellular and Molecular Neuroscience |
| 2805 | Cognitive Neuroscience |
| 2806 | Developmental Neuroscience |
| 2807 | Endocrine and Autonomic Systems |
| 2808 | Neurology |
| 2809 | Sensory Systems |
| Nursing | |
| 2900 | General Nursing |
| 2901 | Nursing (miscellaneous) |
| 2902 | Advanced and Specialized Nursing |
| 2903 | Assessment and Diagnosis |
| 2904 | Care Planning |
| 2905 | Community and Home Care |
| 2906 | Critical Care Nursing |
| 2907 | Emergency Nursing |
| 2908 | Fundamentals and skills |
| 2909 | Gerontology |
| 2910 | Issues, ethics and legal aspects |
| 2911 | Leadership and Management |
| 2912 | LPN and LVN |
| 2913 | Maternity and Midwifery |
| 2914 | Medical–Surgical Nursing |
| 2915 | Nurse Assisting |
| 2916 | Nutrition and Dietetics |
| 2917 | Oncology (nursing) |
| 2918 | Pathophysiology |
| 2919 | Pediatrics |
| 2920 | Pharmacology (nursing) |
| 2921 | Pshychiatric Mental Health |
| 2922 | Research and Theory |
| 2923 | Review and Exam Preparation |
| Pharmacology, Toxicology and Pharmaceutics | |
| 3000 | General Pharmacology, Toxicology and Pharmaceutics |
| 3001 | Pharmacology, Toxicology and Pharmaceutics (miscellaneous) |
| 3002 | Drug Discovery |
| 3003 | Pharmaceutical Science |
| 3004 | Pharmacology |
| 3005 | Toxicology |
| Psychology | |
| 3200 | General Psychology |
| 3201 | Psychology (miscellaneous) |
| 3202 | Applied Psychology |
| 3203 | Clinical Psychology |
| 3204 | Developmental and Educational Psychology |
| 3205 | Experimental and Cognitive Psychology |
| 3206 | Neuropsychology and Physiological Psychology |
| 3207 | Social Psychology |
| Social Sciences | |
| 3306 | Health (social science) |
| Dentistry | |
| 3504 | Oral Surgery |
| Health Professions | |
| 3600 | General Health Professions |
| 3601 | Health Professions (miscellaneous) |
| 3603 | Complementary and Manual Therapy |
| 3604 | Emergency Medical Services |
| 3605 | Health Information Management |
| 3606 | Medical Assisting and Transcription |
| 3607 | Medical Laboratory Technology |
| 3608 | Medical Terminology |
| 3609 | Occupational Therapy |
| 3610 | Optometry |
| 3611 | Pharmacy |
| 3612 | Physical Therapy, Sports Therapy and Rehabilitation |
| 3613 | Podiatry |
| 3614 | Radiological and Ultrasound Technology |
| 3615 | Respiratory Care |
| 3616 | Speech and Hearing |
