# Supplementary material for: Making clinician-scientists visible: methods for identifying clinician research participation and metrics at scale
Source: Front Health Serv. 2026 Apr 30;6:1791235. doi: 10.3389/frhs.2026.1791235 (PMC13171505; doi:10.3389/frhs.2026.1791235)
Supplement: Supplementary file 2 [file Supplementaryfile2.docx]

## Supplementary Two: Example research questions

#### Clinician Research Participation and Metrics

- What is the extent of research participation among registered medical practitioners in Australia, as indicated by verified Scopus author profiles?
- Which types and formats of research outputs (e.g., journal articles, reviews, clinical trials) are most commonly produced by Australian physician-scientists, and how do these relate to their research metrics?
- Which characteristics of Australian medical practitioners (e.g., specialty, qualification type, registration type, years since registration) are associated with greater likelihood of participating in research or achieving stronger research metrics?
- To what extent are newly registered Australian medical practitioners engaging in research, and how do their research metrics compare to those of more experienced cohorts?
- Which combinations of practitioner and contextual characteristics are associated with research participation and stronger research metrics among Australian physician-scientists?

#### Bibliometric Productivity and Impact

- What is the distribution and trajectory of publication counts among Australian physician-scientists (e.g., first vs. most recent publication year)?
- What is the prevalence of first-authorship among publications by Australian physician-scientists, and how does it relate to research performance?
- How do citation-based metrics (e.g., total citations, average citations, h-index) vary by specialty, career stage, or practice setting among Australian physician-scientists?
- To what extent are Australian physician-scientists producing open access publications, and how does this influence citation rates?
- What proportion of Australian physician-scientists demonstrate sustained research output (e.g., more than five publications) or achieve benchmark h-indices (e.g., >5 or >10)?
- What is the relationship between document count and citation count across the cohort of Australian physician-scientists?
- Which types of publications (e.g., clinical trials, reviews) by Australian physician-scientists are most highly cited?
- Disciplinary Focus and Subject Area Trends
- Which ASJC subject areas are most commonly represented in the research outputs of Australian physician-scientists?
- Which ASJC subject areas associated with Australian physician-scientists show the highest average citation impact?
- How are research domains distributed across different medical specialties within the Australian physician-scientist cohort?
- Are there observable interdisciplinary patterns in subject areas represented in Australian physician-scientist publications?
- Are certain ASJC subject areas disproportionately associated with specific groups of Australian medical practitioners?
- Which medical specialties among Australian practitioners contribute across a broader array of ASJC subject areas?

#### Institutional and Geographic Trends

- Which Australian states, cities, or regions have higher concentrations of medical practitioners participating in research?
- What are the most frequently reported institutional affiliations of Australian physician-scientists?
- To what extent do citation metrics vary by geographic location and institutional affiliation among Australian physician-scientists?
- How does practice location (e.g., state, suburb, postcode) relate to research participation and research metrics among Australian medical practitioners?
- What are the patterns of institutional affiliation changes among Australian physician-scientists, and how do these affect research productivity?
- To what extent are Australian clinicians based in hospitals or health services underrepresented in bibliometric outputs?
- How do the research metrics of Australian-trained medical practitioners compare to those trained overseas?
- Which Australian medical programs or universities are most associated with the production of physician-scientists?

#### Knowledge Translation and Non-Traditional Impact

- To what extent are publications by Australian physician-scientists cited in policy documents, media, blogs, or social media?
- Which types of documents authored by Australian physician-scientists generate the most online engagement, and how does this relate to research metrics?
- How does non-traditional impact (e.g., mentions, usage metrics) correlate with traditional citation metrics among Australian physician-scientists?
- Which specialties within the Australian medical workforce are associated with higher levels of non-traditional impact?
- What proportion of publications by Australian physician-scientists demonstrate non-traditional impact in the absence of formal citations?
- How does open access status influence online engagement with research outputs by Australian physician-scientists?

#### Equity and Visibility Gaps

- Which demographic or geographic groups of Australian medical practitioners (e.g., CALD, women, rural/regional) are underrepresented among physician-scientists?
- Are there observable disparities in research metrics or online impact among Australian physician-scientists based on demographic or geographic characteristics?
- Do citation patterns differ by region or specialty among Australian physician-scientists, independent of publication volume?
- How does bibliometric visibility compare across metropolitan and non-metropolitan physician-scientists in Australia?

#### Collaboration and Career Trajectory Trends

- What is the average number of co-authors per Australian physician-scientist, and how does this relate to research productivity and metrics?
- To what extent do high co-author counts among Australian physician-scientists reflect interdisciplinary or multi-institutional collaboration?
- Do early-career Australian physician-scientists tend to publish with consistent co-author networks, and how does this shape their research trajectory?
- What are the dominant patterns of collaboration (e.g., local, national, international) among Australian physician-scientists?
- How does language or cultural diversity among Australian physician-scientists intersect with collaboration networks and research impact?
