# Supplementary material for: Making clinician-scientists visible: methods for identifying clinician research participation and metrics at scale
Source: Front Health Serv. 2026 Apr 30;6:1791235. doi: 10.3389/frhs.2026.1791235 (PMC13171505; doi:10.3389/frhs.2026.1791235)

## Supplementary Three: AHPRA application form—Registration as a medical specialist with no existing general registration
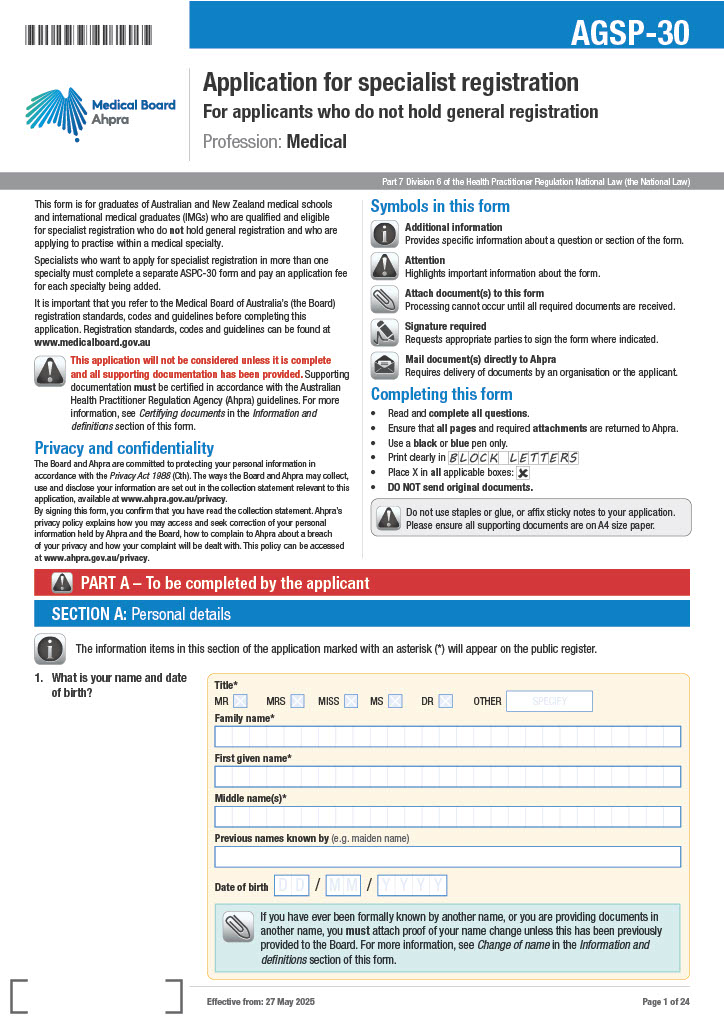

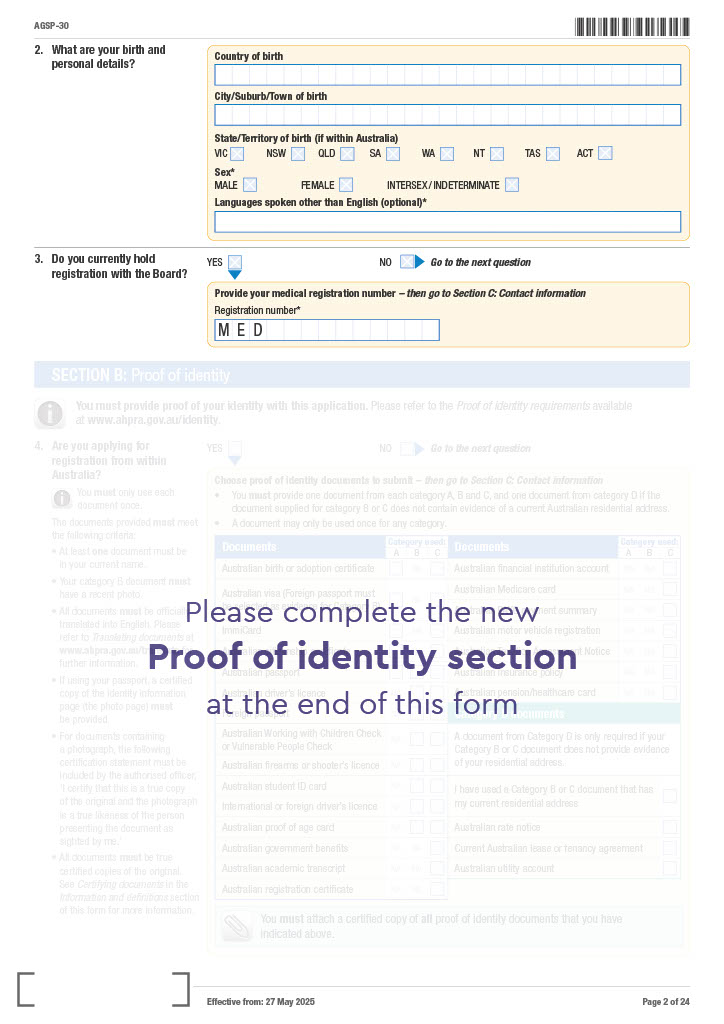

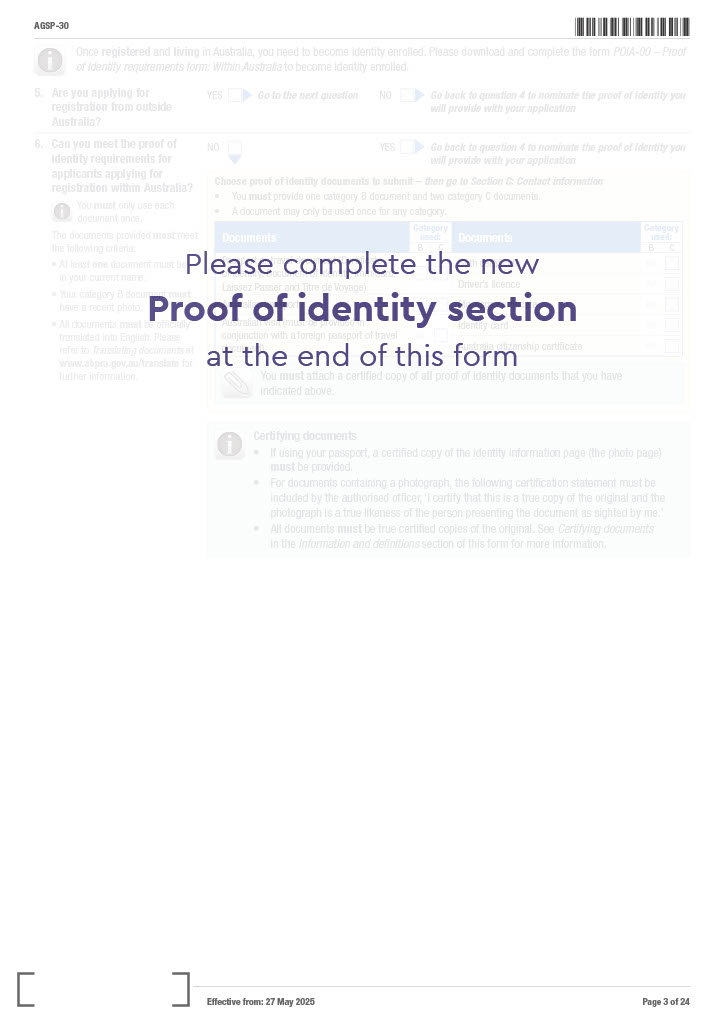

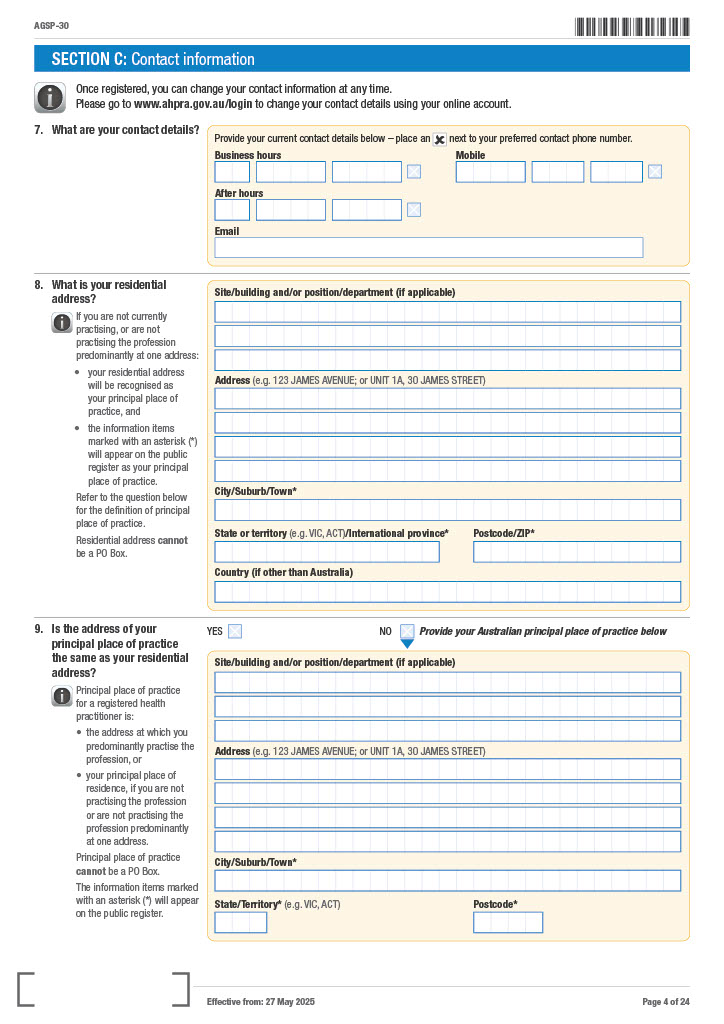

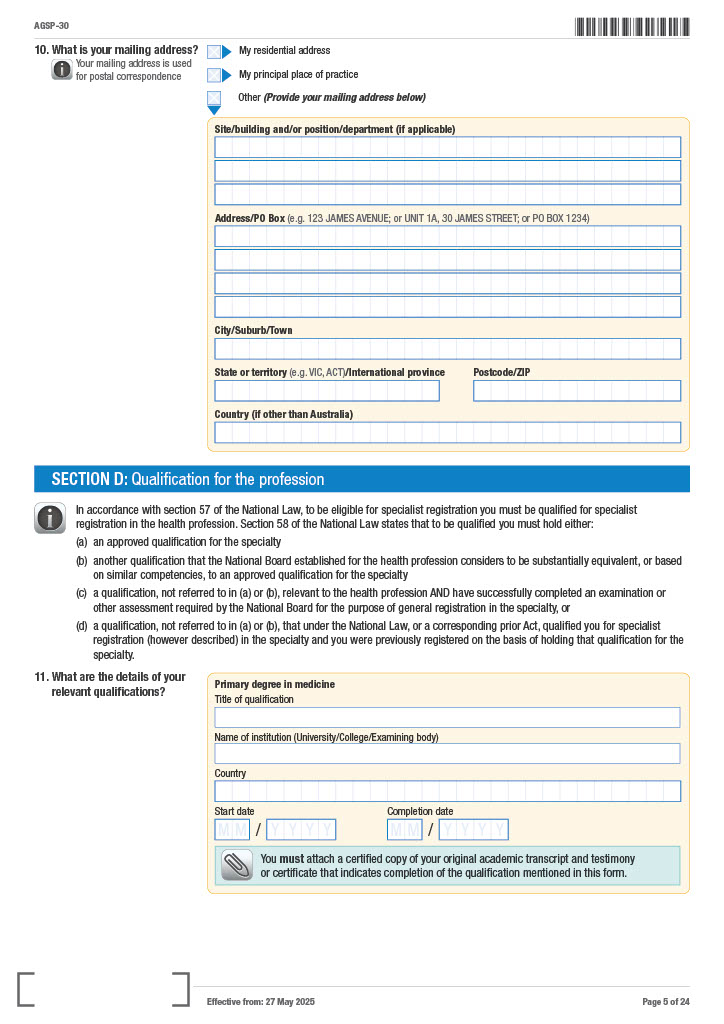

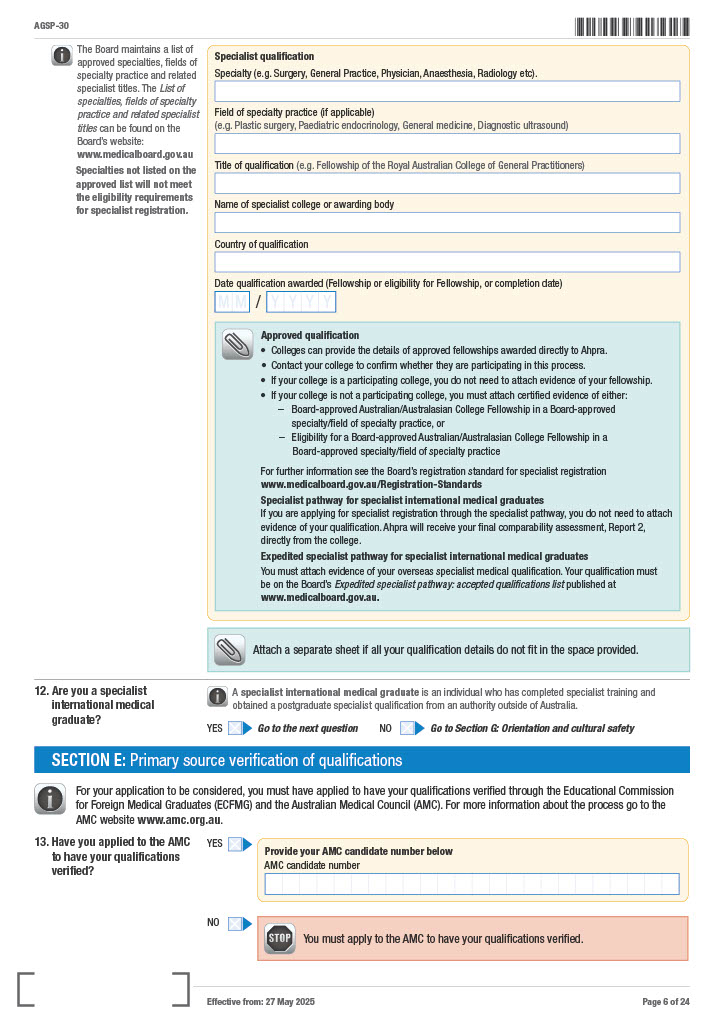

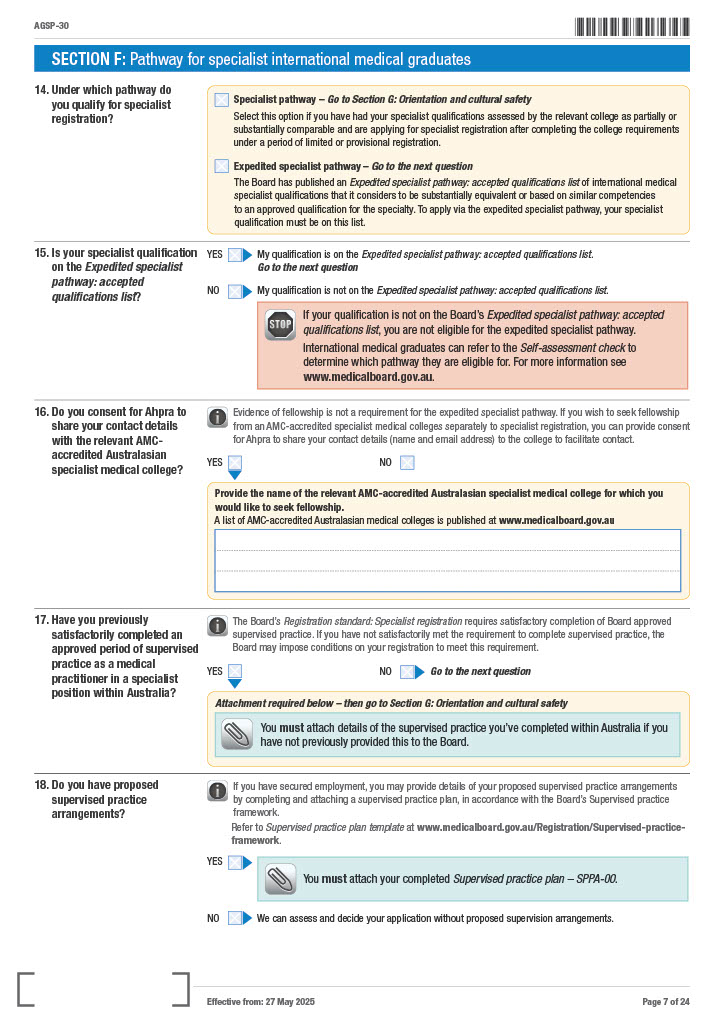

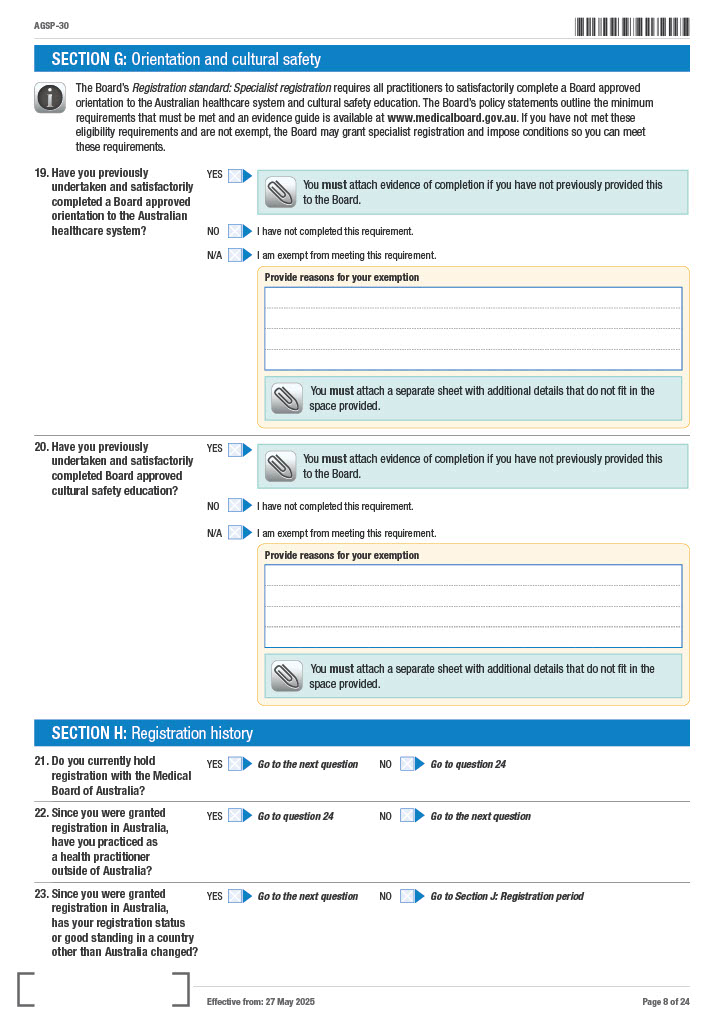

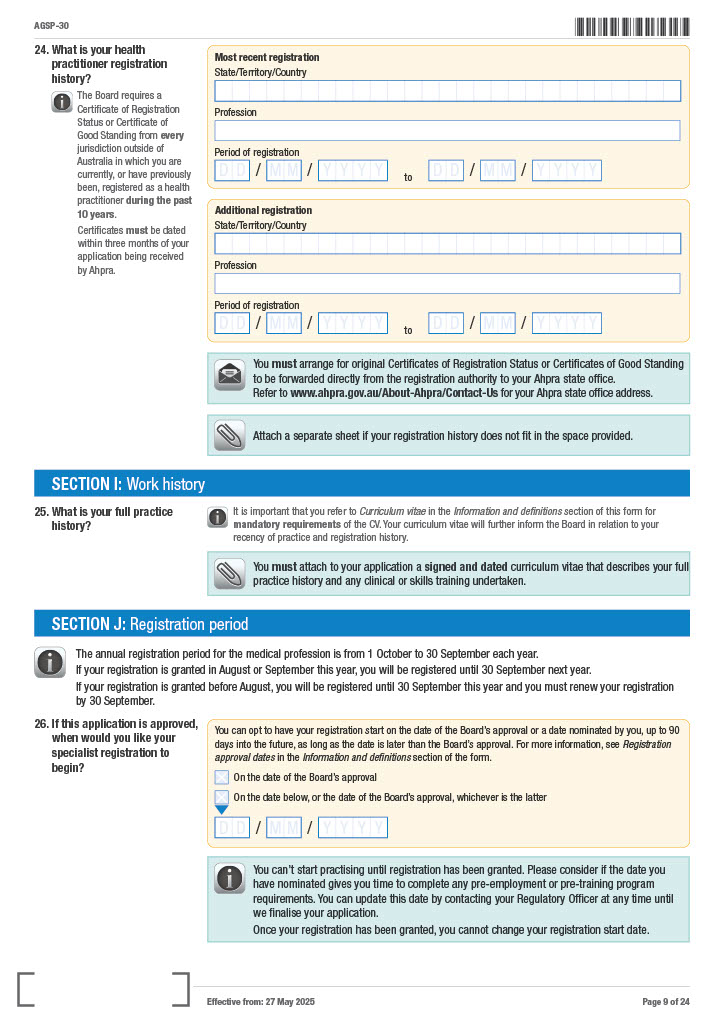

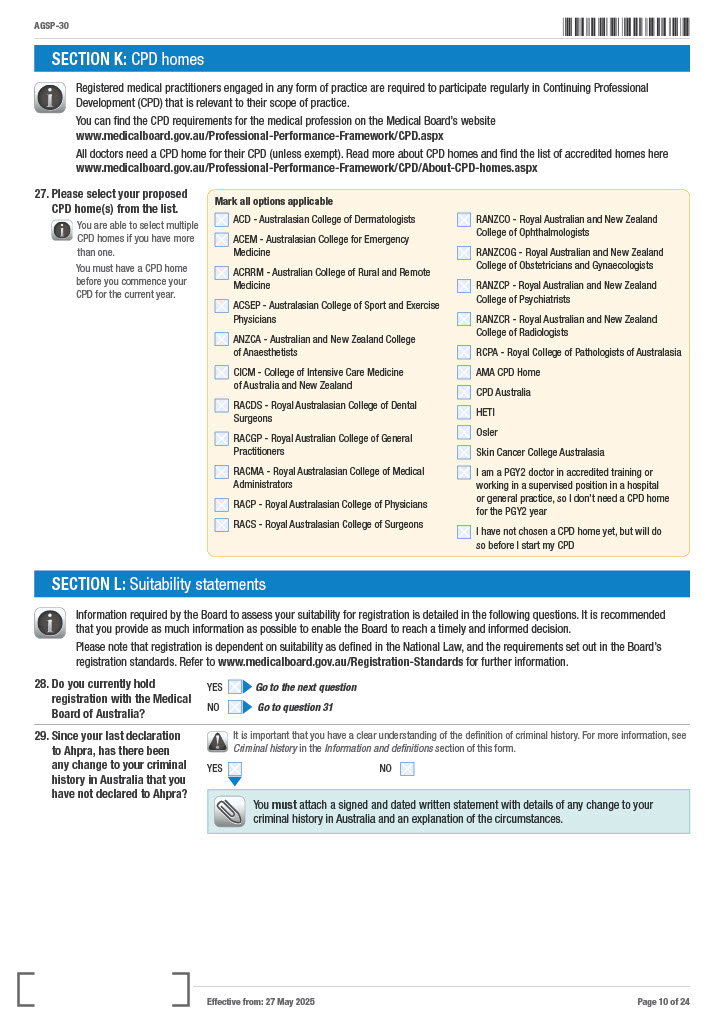

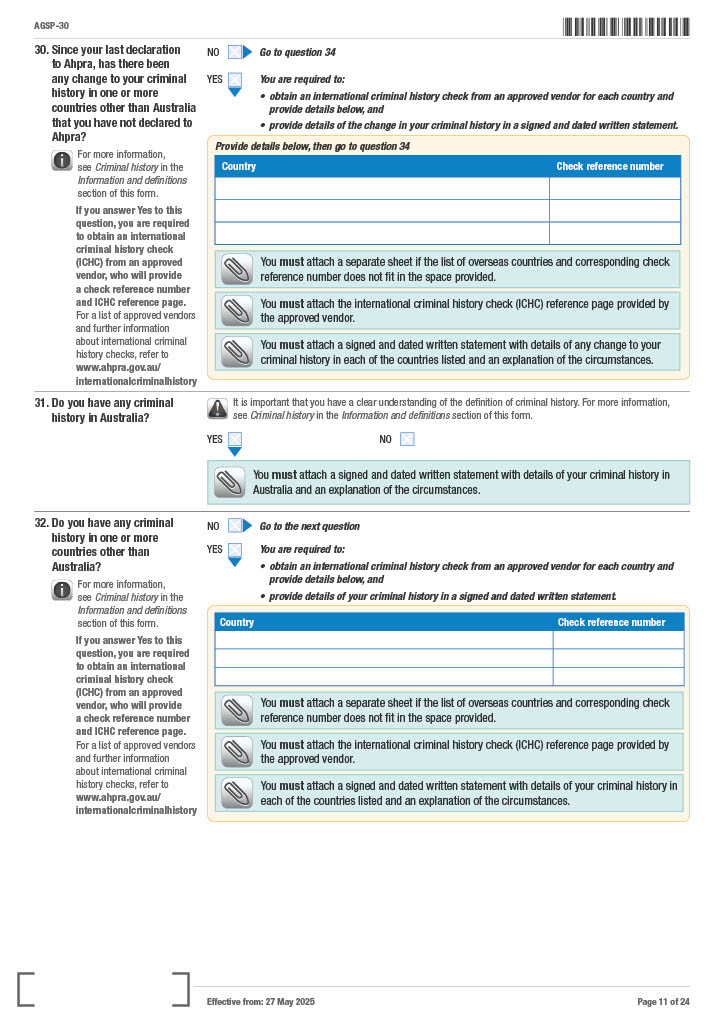

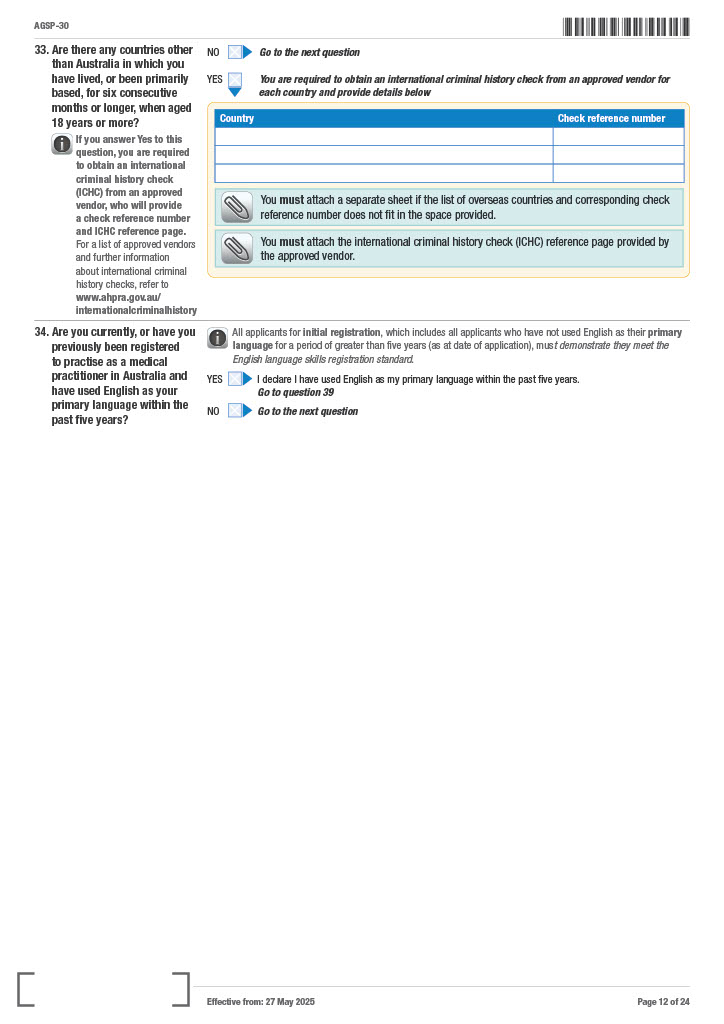

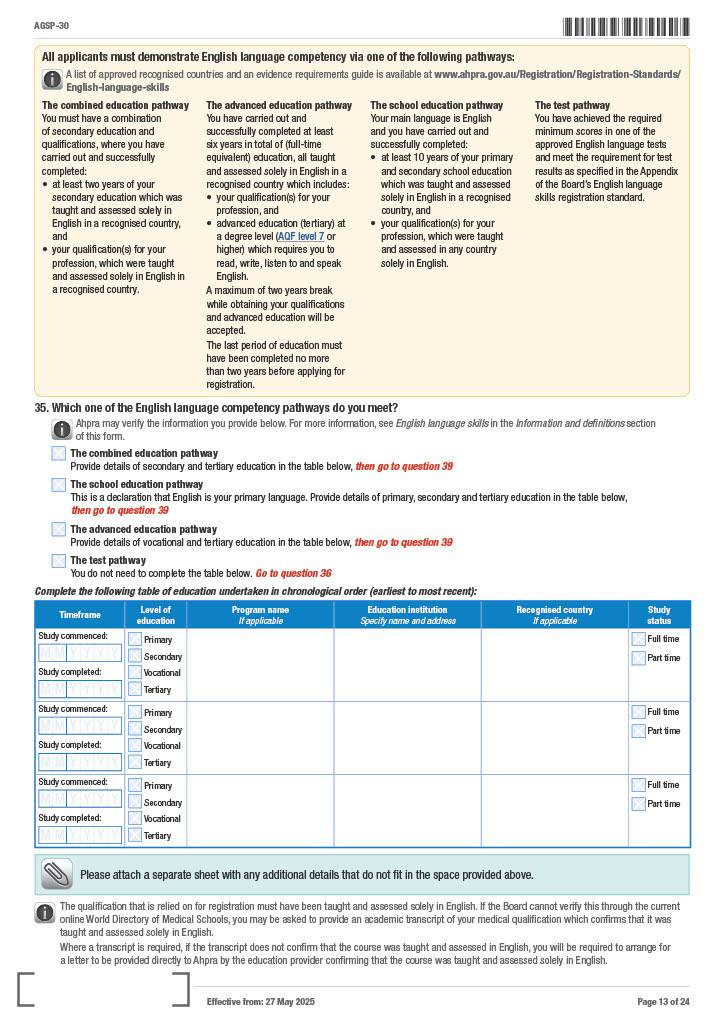

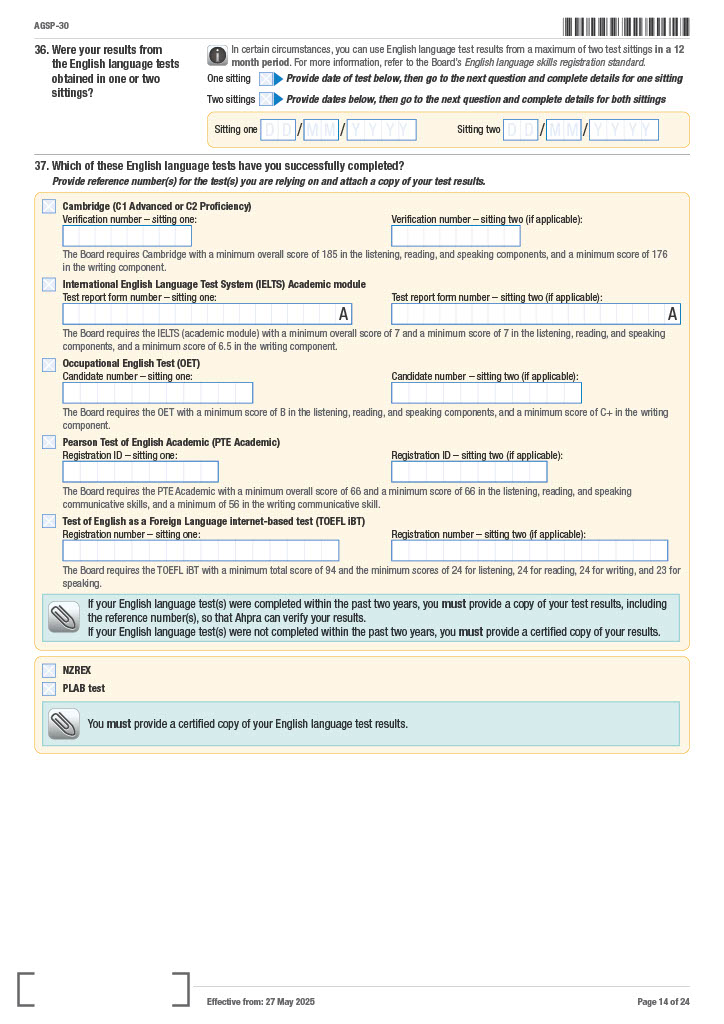

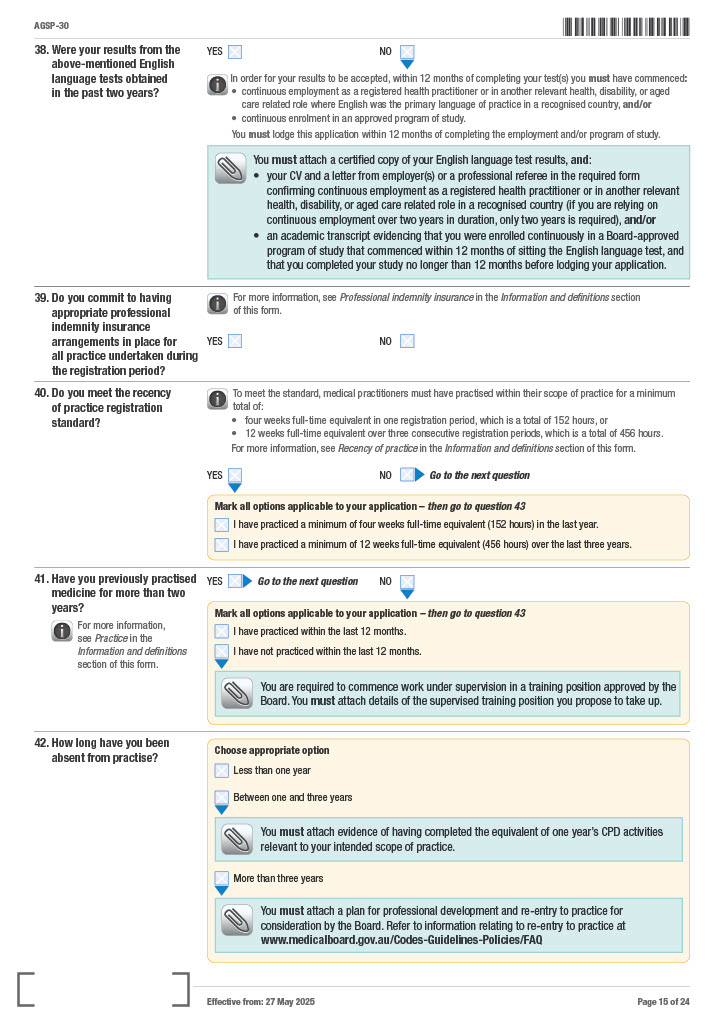

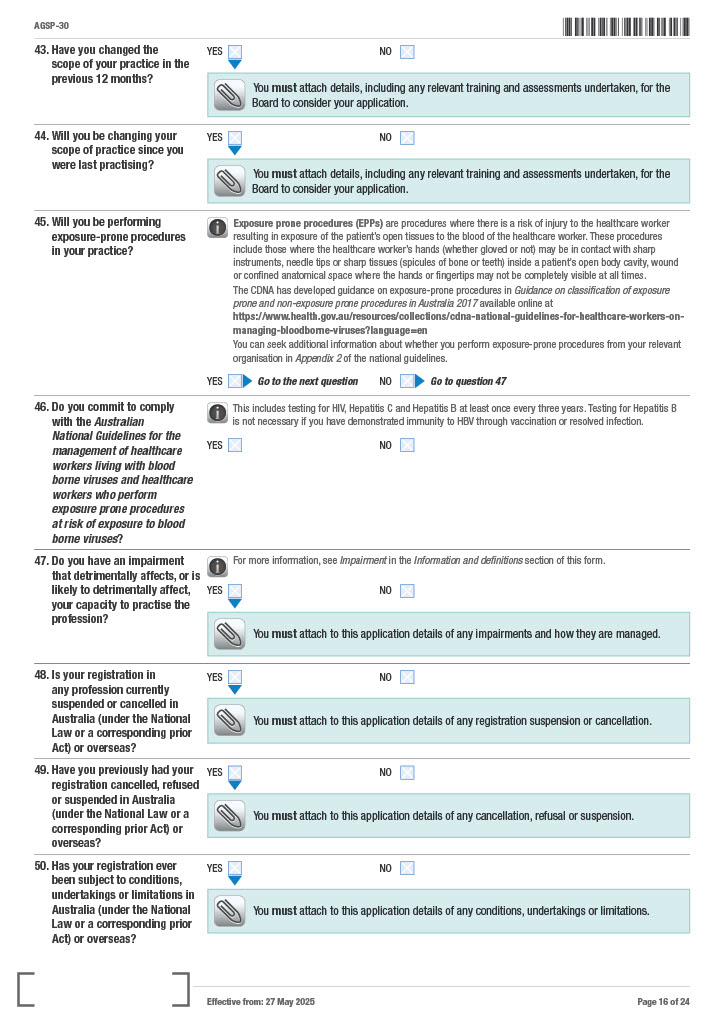

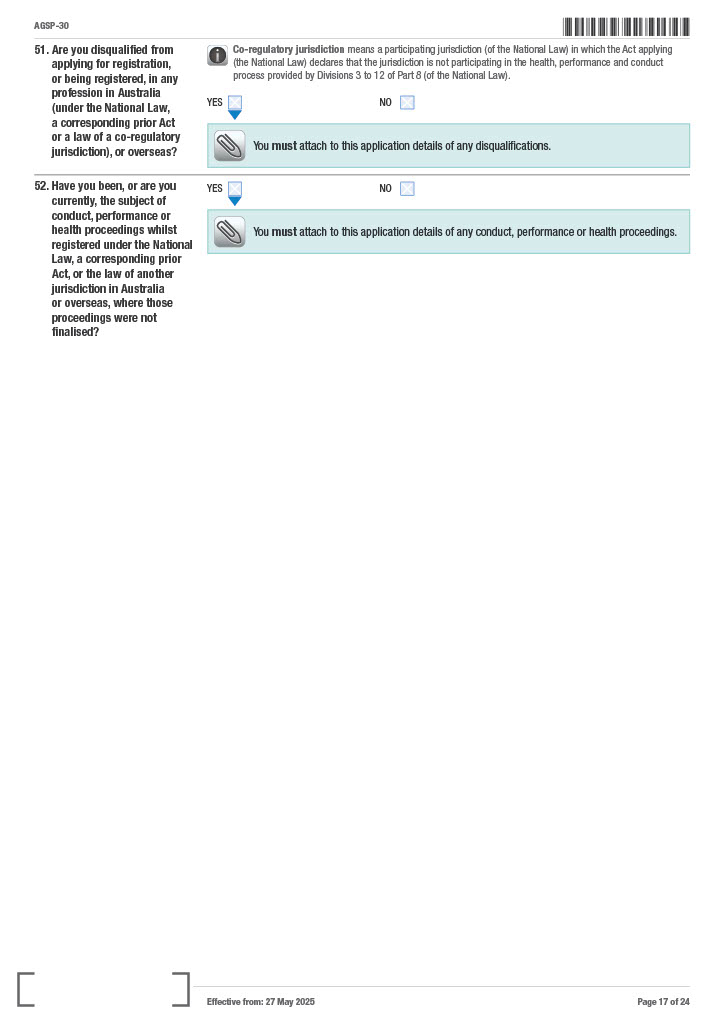

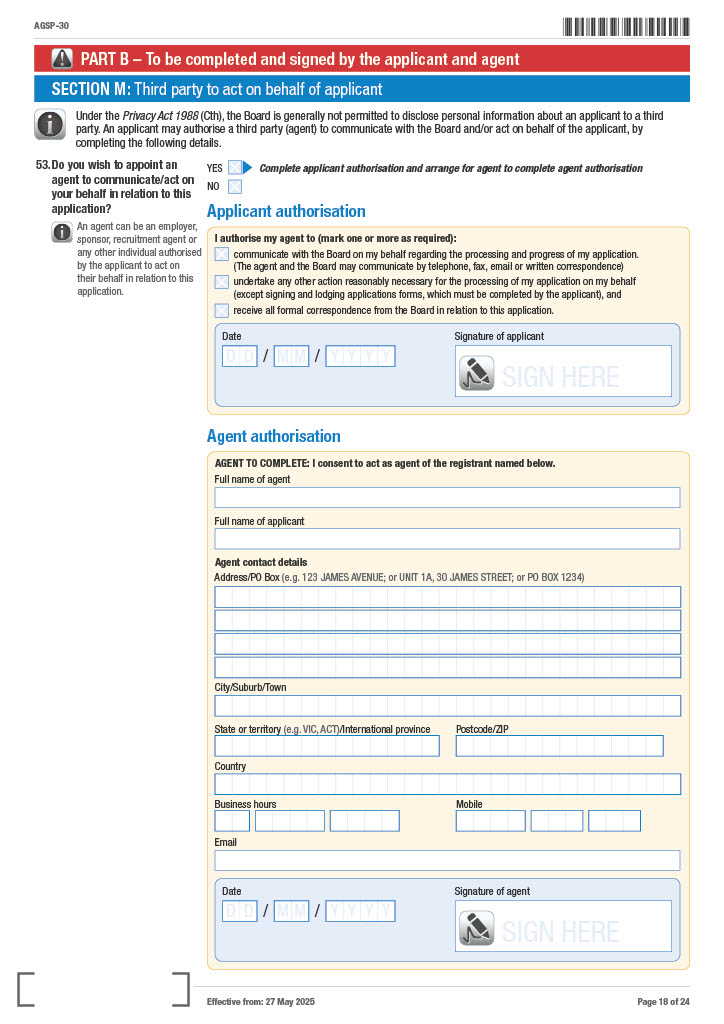

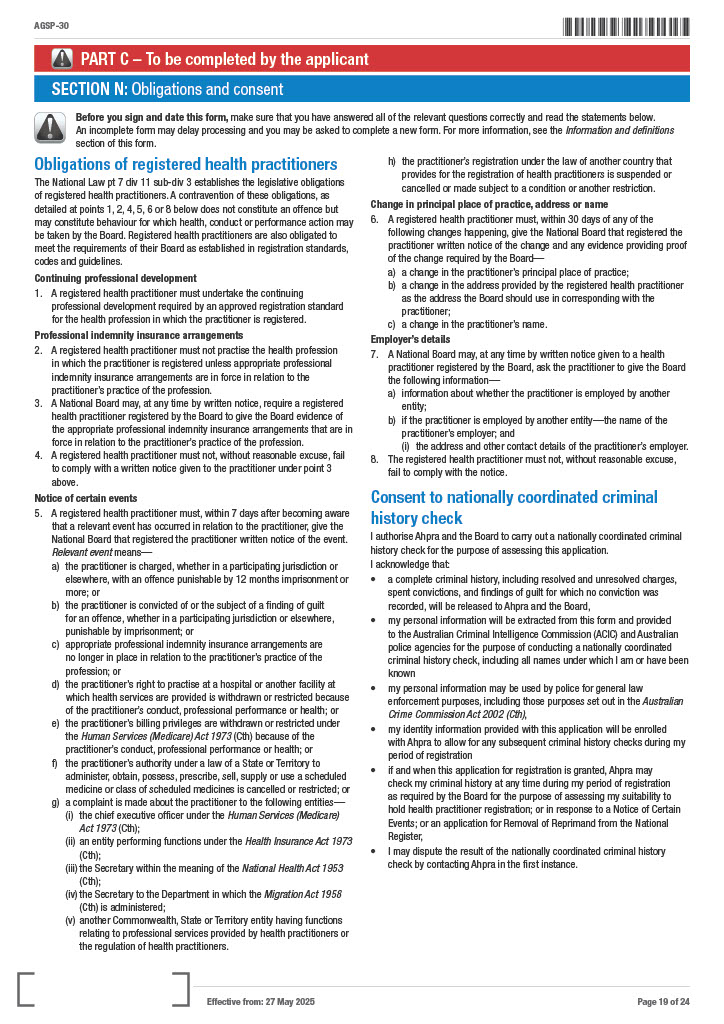

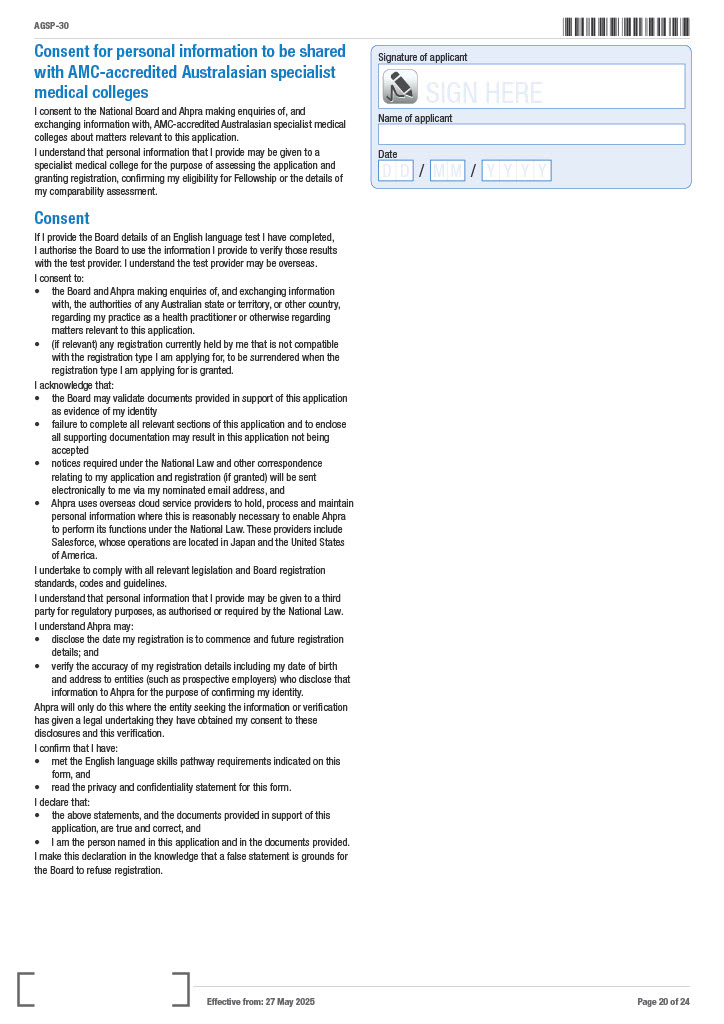

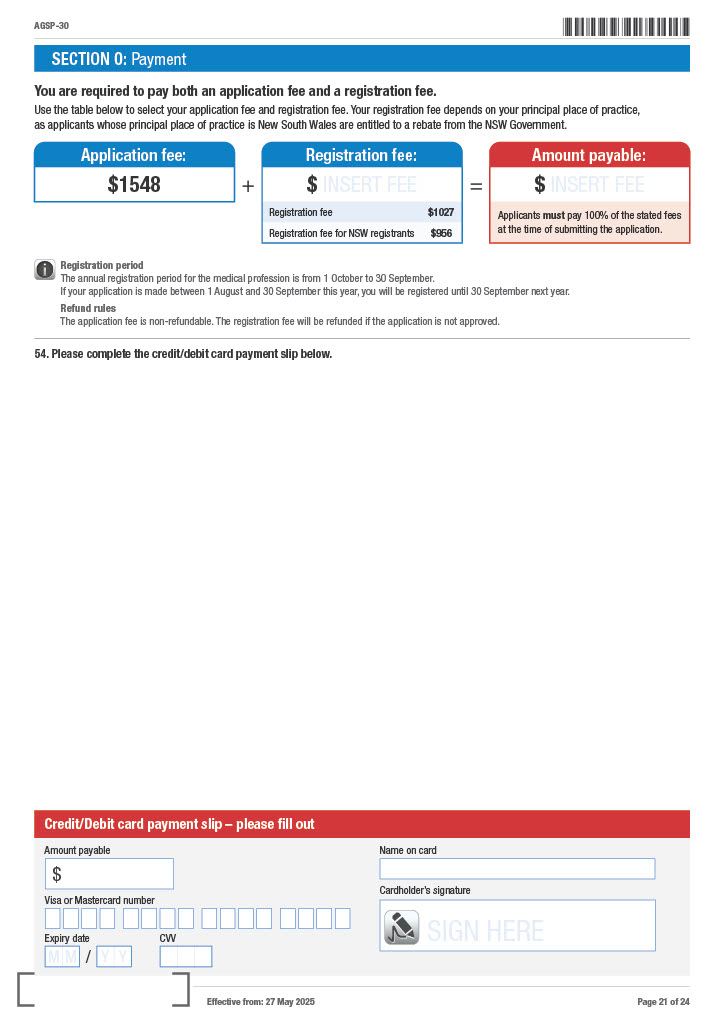

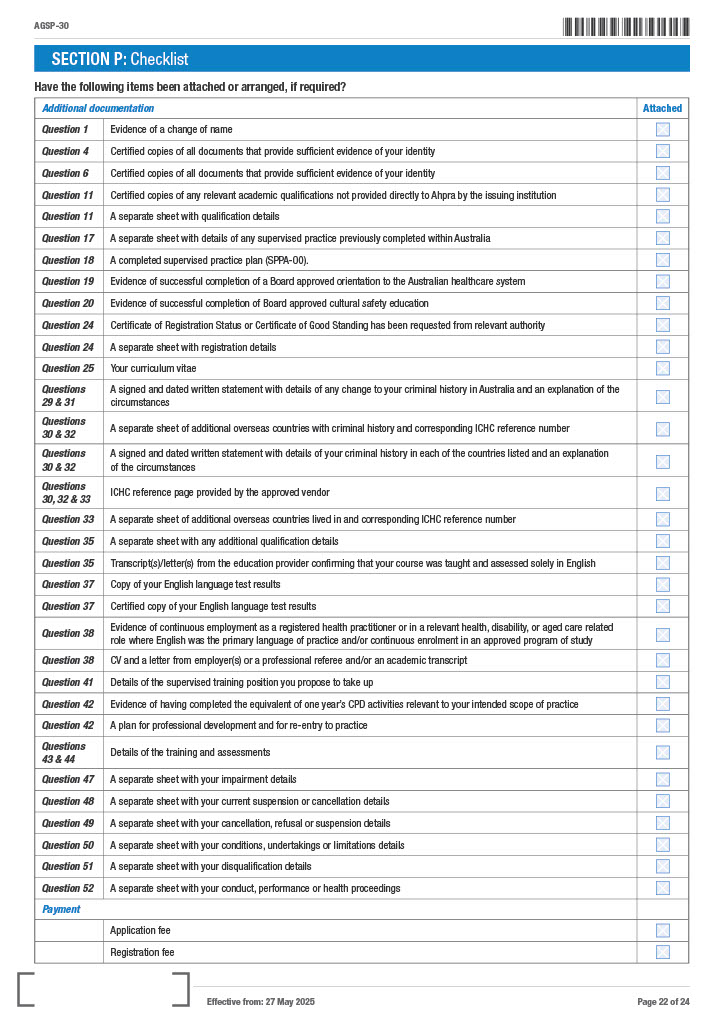

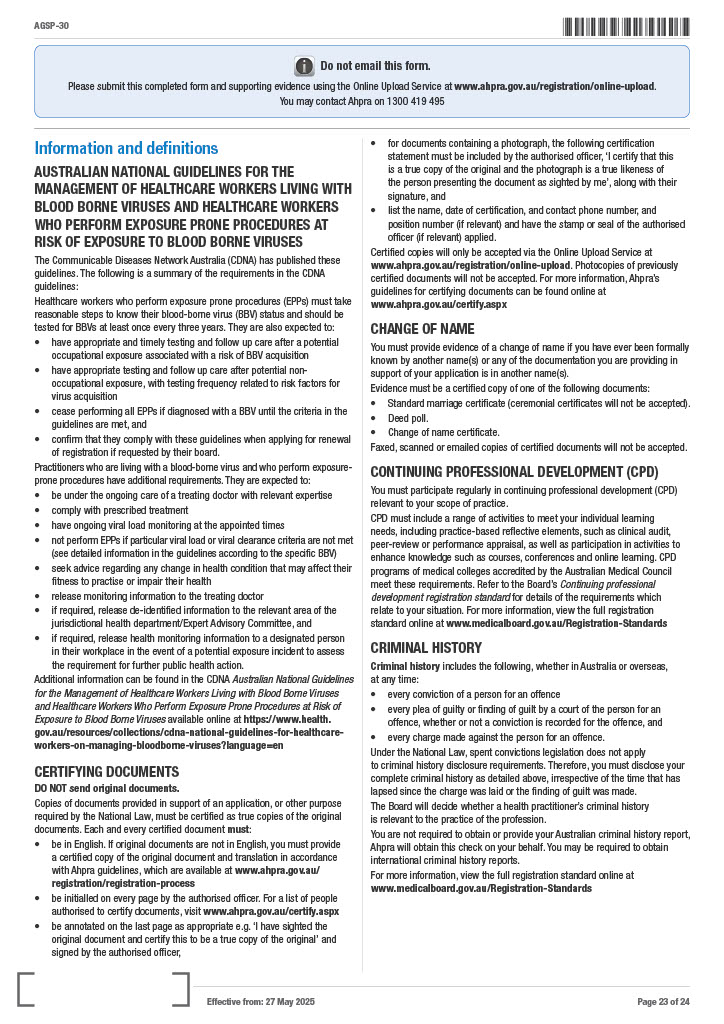

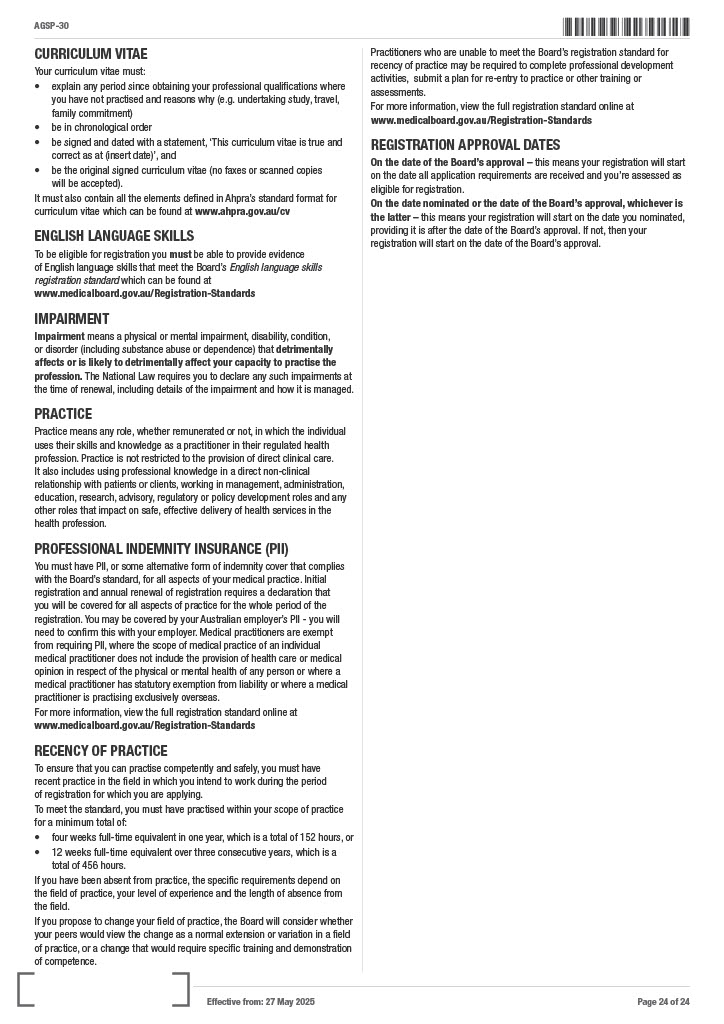

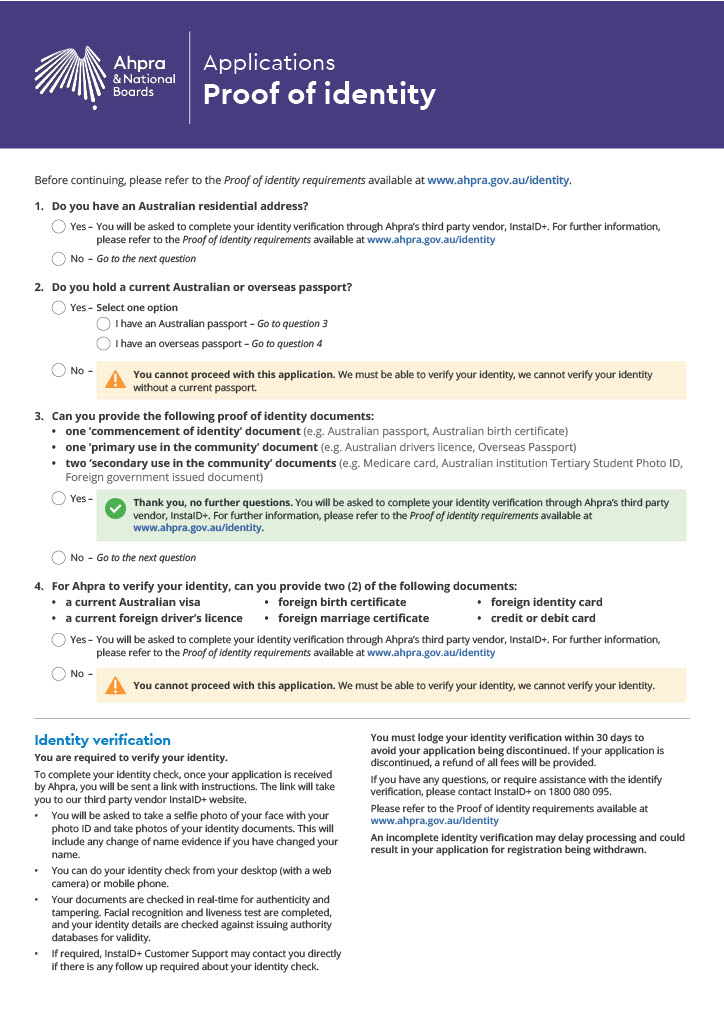

Supplement: Supplementary file 3 [file Supplementaryfile3.docx]
